# Geography is essential for reproductive isolation between florally diversified morning glory species from Amazon canga savannahs

**DOI:** 10.1038/s41598-019-53853-4

**Published:** 2019-12-02

**Authors:** Elena Babiychuk, Juliana Galaschi Teixeira, Lourival Tyski, José Tasso Felix Guimaraes, Luiza Araújo Romeiro, Edilson Freitas da Silva, Jorge Filipe dos Santos, Santelmo Vasconcelos, Delmo Fonseca da Silva, Alexandre Castilho, José Oswaldo Siqueira, Vera Lucia Imperatriz Fonseca, Sergei Kushnir

**Affiliations:** 1 Instituto Tecnológico Vale, Rua Boaventura da Silva 955, CEP 66055-090 Belém, Pará Brazil; 2 Museu Paraense Emílio Goeldi, Departamento de Botânica, CEP 66040-170 Belém, Pará Brazil; 3 Parque Zoobotânico Vale, VALE S.A., Rod. Raimundo Mascarenhas, Km 26, S/N., Núcleo Urbano de Carajás, CEP 68516-000 Parauapebas, Pará Brazil; 4 Gerência de Meio Ambiente, Departamento de Ferrosos Corredor Norte, Vale S.A., Rua Guamá n 60, Núcleo Urbano, CEP 68516-000 Parauapebas, Pará Brazil; 5 Unaffiliated, Belém, Pará Brazil; 6 Present Address: Teagasc, Crop Science Department, Oak Park, Carlow, R93 XE12 Ireland

**Keywords:** Evolutionary ecology, Speciation

## Abstract

The variety, relative importance and eco-evolutionary stability of reproductive barriers are critical to understanding the processes of speciation and species persistence. Here we evaluated the strength of the biotic prezygotic and postzygotic isolation barriers between closely related morning glory species from Amazon canga savannahs. The flower geometry and flower visitor assemblage analyses supported pollination by the bees in lavender-flowered *Ipomoea marabaensis* and recruitment of hummingbirds as pollinators in red-flowered *Ipomoea cavalcantei*. Nevertheless, native bee species and alien honeybees foraged on flowers of both species. Real-time interspecific hybridization underscored functionality of the overlap in flower visitor assemblages, questioning the strength of prezygotic isolation underpinned by diversification in flower colour and geometry. Interspecific hybrids were fertile and produced offspring in nature. No significant asymmetry in interspecific hybridization and hybrid incompatibilities among offspring were found, indicating weak postmating and postzygotic isolation. The results suggested that despite floral diversification, the insular-type geographic isolation remains a major barrier to gene flow. Findings set a framework for the future analysis of contemporary evolution of plant-pollinator networks at the population, community, and ecosystem levels in tropical ecosystems that are known to be distinct from the more familiar temperate climate models.

## Introduction

The natural world biodiversity is recognizably discontinuous^1^. Gaps in morphology often separate groups of organisms that we call species^2–4^. In sexually reproducing organisms, the occurrence and evolvement of reproductive barriers, counteracting the blending effects of gene flow, are thought to be essential for the evolutionary independence of populations^5^. In plants, 70% of taxonomic species and 75% of phenotypic clusters correspond to reproductively independent lineages, showing that reproductive isolation is an essential cause of plant biodiversity^6^. Thus, understanding reproductive isolation remains a central question in studies focused on the origin of species, process of speciation^7–9^.

The formation of a zygote is a key chronological landmark to distinguish reproductive barriers acting before and after successful fertilization, the so-called prezygotic and postzygotic isolation^8^. Prezygotic isolation mechanisms are commonly split into premating and postmating isolation. Premating, prepollination barriers in plants are exemplified by habitat isolation^10,11^, phenological differences, e.g. differences in flowering time^11^, and pollinator specificity^12^. Events happening after pollen grain deposition at a flower stigma surface constitute postmating or else known as postpollination isolation barriers in plants, which comprise pollen-pistil incompatibilities and congeneric pollen disadvantages^9,13^. Postmating barriers underpinned by gametic competition are documented in birds^14^, fish^15^ and insects^16^. Reduced hybrid viability and infertility are two major phenotypic manifestations of postzygotic isolation both in plants and animals^7–9,17^. The genetic architecture of postzygotic isolation is explained by the Bateson–Dobzhansky–Muller genetic incompatibilities^18^ and epigenetic variation^19^. In plants, presence-absence variation among duplicated gene copies can cause recessive embryo lethality^20^ and sterility^21^. Autoimmune responses due to epistatic interactions between natural genetic variants lead to common hybrid necrosis^22^. Structural genome variation, such as translocations and chromosome copy number disparity are the common causes of meiosis abnormalities that abort plant and animal gametogenesis, underpinning various degrees of infertility^23–25^, although plant meiosis seems to be lacking in the pachytene checkpoint control^25^ and genic incompatibilities^26^ could be more common causes pollen unviability^27^. Chromosomal inversions can play a role in local adaptation and diversification, for example in butterflies^28^ and monkeyflowers (*Mimulus*)^9^. One defining characteristic of a metazoan species can be a coadapted mitonuclear genotype that is incompatible with the coadapted mitochondrial and nuclear genotype of other populations^29^. Intracellular coadaptation between the nuclear and cytoplasmic genomes influences both viability and fertility of plant hybrids^9^.

Although plants vary in patterns of reproductive isolation, studies of species from twenty-one genera suggested that prepollination barriers were often very strong in plants and contributed more to total reproductive isolation than postpollination and postzygotic barriers^7,8^. The major biotic players in prepollination isolation are thought to be pollinators^12^. Sexual reproduction in 87% of flowering plants is dependent on animals for pollen transfer^30^. In a basic scenario, animals are attracted by the flower shape, colour, scent, and rewarded with nectar and pollen as a food^31–34^. To function in plant reproductive isolation, mutualistic plant-pollinator interaction must show pairwise specificity. The strong innate pollinator preferences in colour, spatial achromatic flower properties, or scent can underpin mutualism specificity^35–37^. Thus, the concept of pollination syndromes proposes that specific suites of flower traits evolved in response to natural selection imposed by different pollinators^38,39^. The Grant-Stebbins model can explain pollinator-driven floral diversification^40–42^. In this model, geographical differences in pollinator abundance drive adaptive divergence in floral traits across plant populations leading to pollination ecotypes and establishment of a prezygotic reproductive barrier, the so-called floral isolation^12^. Pollinators flexibility in preference due to learning associations between rewards and colour, or due to a variable perception of colour in different environments or plant communities make pollinator imposed selection context-dependent, adding the next layer of sophistication and complexity to floral isolation mechanisms^43,44^. Reward economics is another factor affecting plant-pollinator interactions^45^. For example, genetic analysis in *Mimulus*^44^, ecological studies of Cerrado savannahs plant communities^46^, artificial manipulations of nectar volumes in *Penstemon spectabilis*^45^ all argue that mean nectar offer is the only parameter related to hummingbird visitation frequency, regardless of the flower colour or pollination syndrome^46,47^.

The natural hybrid zones between either currently diversifying populations or at secondary contacts provide evidence for the leakage of prezygotic reproductive barriers. At least 25% of plant species and 10% of animal species are known to hybridize^48^. Depending on the strength and a type of postzygotic isolation, the evolutionary role of interspecific hybrids can have dramatically different consequences. The hybrid sterility can be resolved by polyploidization, which is a typical path in plant allopolyploid speciation^49^. If interspecific hybrids do not suffer severe sterility problems, they can give rise to new homoploid species in plants and birds^50,51^; facilitate genetic rescue and demographic recovery^52,53^; or underpin introgression of favourable traits, the so-called adaptive introgression^54,55^, which explains the evolvement of invasiveness^56^ and recent adaptations to the changing environment, for example in fish^57^. Thus, interspecific introgression can play an essential general role in the process of natural selection by contributing to the generation of intraspecific phenotypic variation, in addition to the *de novo* mutations, recombination, and standing phenotypic variation^55^. At the opposite end of the interspecific hybridization effect spectrum is species extinction by genetic and demographic swamping^58^.

**Figure 1:**
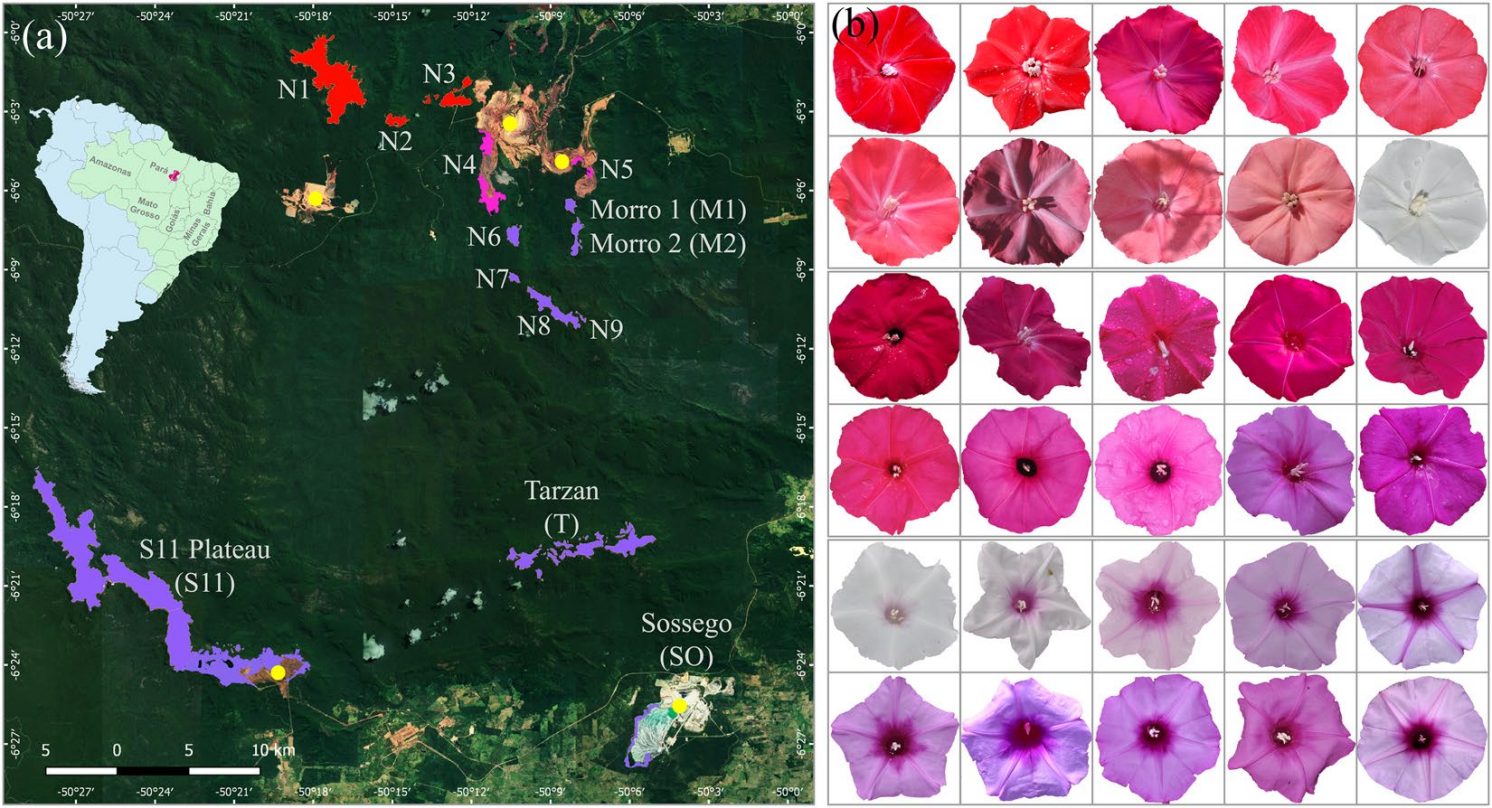
Species geographic distribution and variation in flower colour. (**a**) The map illustrates the study locations. Dark green colour is due to the rain forest that covers eroding Carajás mountain range, the part of which is preserved within the Carajás National Forest. Canga savannahs evolved on iron lateritic rocks of the mountain plateaus that are false coloured in Adobe Photoshop CS6 to emphasize the morning glory species distribution. The allopatric *I. cavalcantei* populations are found in canga N1, N2 and N3, which are in red, according to the predominant flower colour of the species. Lavender coloured cangas N6 to N9, Morro 1 (M1) and Morro 2 (M2), Tarzan (T), S11 plateau (S11) host *I. marabaensis* allopatric populations. Magenta colour of the cangas N4 and N5 remains signifies the species co-occurrence, i.e., sympatric cangas. Sossego (SO) is a granitic inselberg populated by *I. marabaensis* where the species grows along the boundaries of exposed granitic bedrocks and the forest. Insert: red pin on a map of Latin America indicates the location of the Carajás National Forest. Yellow dots point the open pit mines. Cangas are named in accordance with the geological survey maps. The geographic map was generated with the software QGIS version 2.18 (http://qgis.org) based on satellite imagery source (https://mt1.google.com/vt/lyrs%3Ds%26×%3D%7Bx%7D%26 y%3D%7By%7D%26z%3D%7Bz%7D&zmax = 20&zmin = 0) from Google (Google Maps satellite Carajás, Pará, Brazil; retrieved December 16, 2018). (**b**) Variation of flower colour. Two upper rows of flower image series show colour variation within *I. cavalcantei* populations. The two uppermost images to the left represent a typical intense red colour of the species flowers. The following flowers illustrate colour deviations, including pure white, and these were found in sympatric cangas N4, N5 and in allopatric N3. The plants growing in the most distant canga N1 develop red flowers. Flowers of putative hybrids from sympatric canga N4 and N5 had unusual colours of intense magenta, red, pink and purple as shown in two rows of images in the middle. Flower series of *I. marabaensis* are in the two lowest rows. The intensity and patterning of *I. marabaensis* flower limb colours are variable. The most common flower limb colour in all cangas is a light lavender as in the rightmost image at the bottom. *I. marabaensis* plant with pure white flowers was found in sympatric N4. Flower images are not on the same scale.

Here, we determine how reproductive isolation and interspecific hybridization contribute to the evolution of *Ipomoea cavalcantei*^59^ and *I. marabaensis*^60^ morning glory species that inhabit Amazon savannah-like ecosystems known as cangas^61^. This system has several advantages that allow selection and gene flow to be analysed in nature. Firstly, canga evolved on iron laterite rock outcrops found at some isolated mountain tops in Carajás mountain range at the similar elevations of ca. 700 m above sea level; are surrounded by a very dense mountainous rain forest, the canopy of which towers at 10–20 meters, and are often separated by the ravines in the eroding mountainous landscape, indicative of the insular type geographic isolation^62^ (Supplementary Fig. S1). Secondly, species have a largely allopatric distribution between canga islands that are spaced at a relatively small geographic scale^62^ (Fig. 1a). Thirdly, taxonomists proposed that *I. cavalcantei* and *I. marabaensis* are sister species^60^. Our phylogenetic analysis supported close species relationship^62^. Next, possible hybridization zone may exist, as suggested by the reports of individuals with intermediate morphological characteristics^62,63^. Finally, the major interspecific phenotypic differences are flower shape, size and colour, red in *I. cavalcantei* and lavender in *I. marabaensis*^59,60,63^. The blue-to-red flower colour transition is thought to underpin a shift in pollinator specialization from bees to hummingbirds in *Ipomoea*^39,64,65^. In the genus, flowers are predominantly coloured by the pelargonidin-based (red hues) and cyanidin-based (blue-purple hues) anthocyanins^39,66^ and to a lesser extent by carotenoids^67^. The ancestral floral colour is blue/purple^65,68^. Several independent transitions to other colours have occurred in this genus, including at least four red-flowered lineages^68^. The red flower colour in cypress vine morning glory *I. quamoclit* is determined by the down-regulation of the flavonoid 3′ hydroxylase (F3′H) in flower tissues, which redirects metabolic flow towards biosynthesis of pelargonidin^64,65^.

The experimental design of this study tested two hypotheses: (i) floral isolation is an important component of prezygotic isolation between *I. cavalcantei* and *I. marabaensis*; (ii) interspecific hybridization can enhance gene flow that contributes to the generation of phenotypic variation in the *I. cavalcantei* and *I. marabaensis* species complex.

## Results

### Species flowering time overlapped at sympatry and migration sites

We re-evaluated distribution of red-flowered *I. cavalcantei* and lavender-flowered *I. marabaensis* individuals in thirteen canga islands within the Carajás National Forest (Fig. 1a; Supplementary Table S1). Sympatry was found only in cangas N4 and N5. *I. cavalcantei* was common in canga N4, whereas *I. marabaensis* was not abundant and only appeared as groups of 10–20 individuals close to the canga-forest boundaries. It was difficult to compare the historical abundance of *I. cavalcantei* and *I. marabaensis* in canga N5, of which only 9% is available for studies^61^. At two N5 survey sites, both species were intermixed in comparable frequencies and the distance between plants with red and lavender flowers varied between 0.5 to 10 meters (Supplementary Fig. S2), indicating that species were exposed to similar animal pollinator assemblages. A single *I. cavalcantei* migrant surrounded by numerous *I. marabaensis* plants was found in allopatric N8 that is 6-km-distant from sympatric N4 (Supplementary Fig. S2).

To determine the potential role of flowering timing in prezygotic isolation^11^, we followed species for three years. During the dry seasons, perennial morning glories shed leaves and did not flower. During the wet seasons, depending on the time of rainfall, *I. marabaensis* flowering started at the end of January and ended at the end of May or early June. *I. cavalcantei* began flowering at the mid-to-end of February and continuing to the end of May beginning of June. In March and April, both species flowered abundantly at all sympatry (N4, N5) and migration (N8) sites, e.g., Supplementary Fig. S2, indicating a limited role of flowering time differences in reproductive isolation.

### Phenotypic screening reveals variation in flower colour, shape, and size

**Figure 2:**
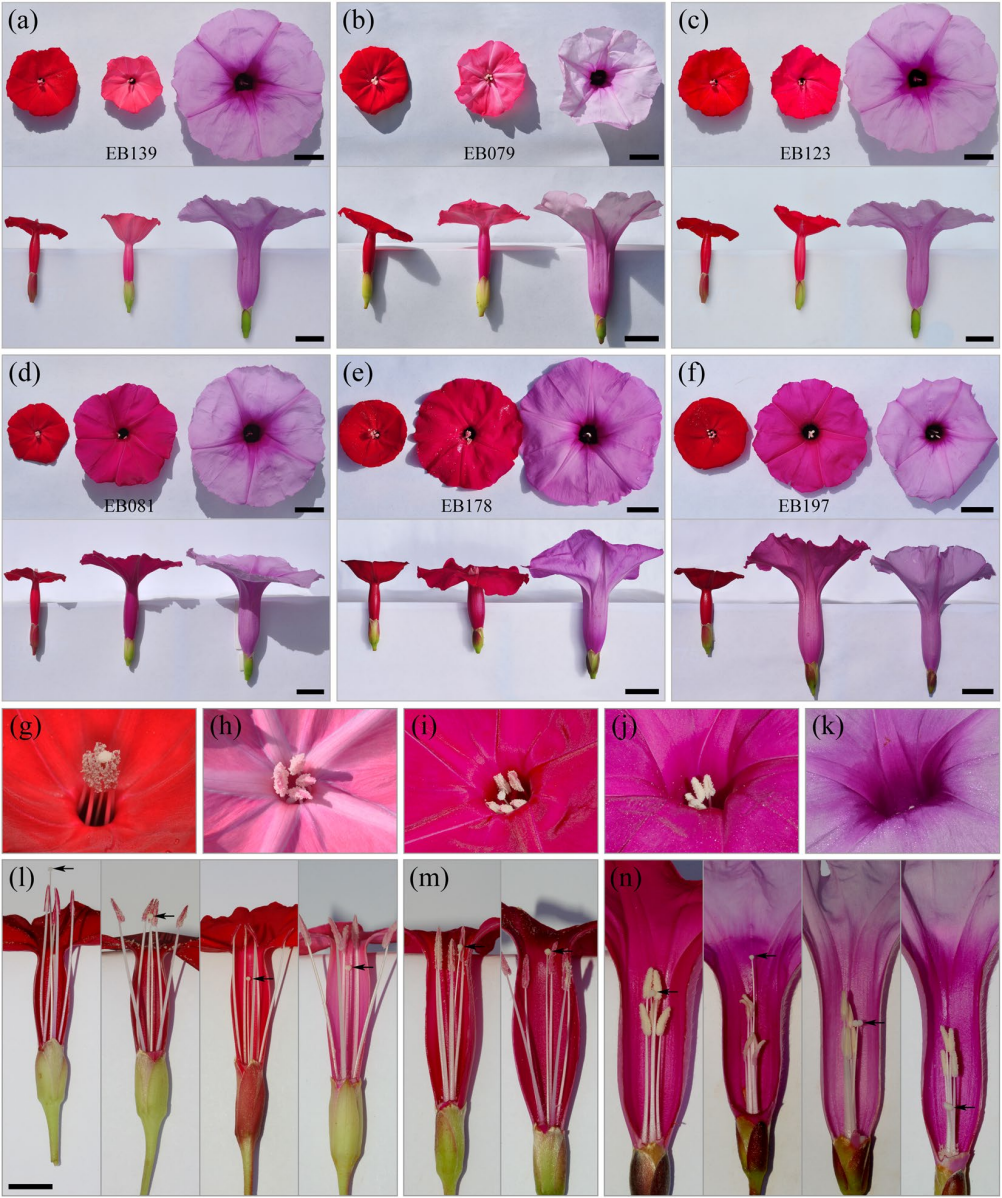
Flower geometry and colour differences. In panels (**a**) to (**f**), flowers of *I. cavalcantei* and *I. marabaensis* are to the left and the right, respectively; bars = 2 cm. In (**g–k**), images of flowers photographed at approximately 45° angle to the flower axis to illustrate reproductive organ positions at the flower throat, not on the same scale. The (**l**–**n**) panels show the range of the stigma positions pointed with black arrows, scale bar = 1 cm in (**l**) applies to (**m,n**). (**a**) Cup-shaped pink flower of line EB139 from canga N4. (**b**) EB079 pink flower (N4) has a longer tube, broader limb, very pale rays - star pattern. (**c**) Purplish flower colour in line EB123, canga N3. (**d**) Flower of a hybrid EB081 from canga N4 has magenta colour. (**e**) EB178 hybrid from canga N5. (**f**) Intense-pink coloured flower of *I. marabaensis*-like line EB197 from canga N5. (**g**) *I. cavalcantei* reproductive organs, anthers supported by filaments, collectively stamens, and style ending with stigma are exserted. (**h**) Close up of EB079 pink flower in (**b**); anthers are relatively low, stigma is included. (**i**) EB081 hybrid. Reproductive organs are included. (**j**) Pink coloured flower of *I. marabaensis*-like line EB197. (**k**) *I. marabaensis* has included styles and stamens that are barely visible at this angle. (**l**) Red *I. cavalcantei* and the rightmost EB079 pink flowers. (**m**) Magenta colour flowers. (**n**) The leftmost intense pink EB197 and *I. marabaensis* flowers. Note that the flower tube insides are intensely coloured in *I. marabaensis*, indicative of the “signpost” patterning guiding visiting pollinators.

To assess the constancy^39^ of the red flower colour in *I. cavalcantei* and identify possible examples of interspecific gene flow effects^69^, populations in canga N1, N2, N3, N4, and N5 were screened for the qualitatively distinguishable colour variation (Fig. 1b). We found seventeen *I. cavalcantei*-like individuals that had pink flowers (cangas N3, N4, N5), e.g., Fig. 2a,b; eleven plants with purplish (N3, N4, N5), e.g., Fig. 2c and two pure white (N3, N4) flower colours. The colour patterning due to lower intensity along the corolla rays that gave a star-like appearance distinguished three pink flowered individuals from sympatric N4 (Figs.. 1b; 2b,h). A similar screen of *I. marabaensis* populations revealed variation in lavender colour patterning and intensity (Fig. 1b). The extreme phenotypical groups included four individuals with white limbs and lavender tubes in allopatric N6; one with pure white flowers in sympatric N4; six with pink flowers in sympatric N5 (Fig. 2f). Variation in flower size and flower limb shape is illustrated in Supplementary Figs. S3 and S4.

Plants with intermediate flower morphological characters hereafter referred to as putative interspecific hybrids (PH), distinguished sympatry cangas N4 (n = 7) and N5 (n = 34). PH plants had intense magenta coloured tubular flowers (Figs.. 1b and 2d). Magenta flower colour in *Ipomoea purga* is due to the production of a mixture of pelargonidin (red) and cyanidin (blue/purple) compounds^39^.

### Coordinated changes in limb contour and throat width had been a major trend of floral shape divergence

**Figure 3:**
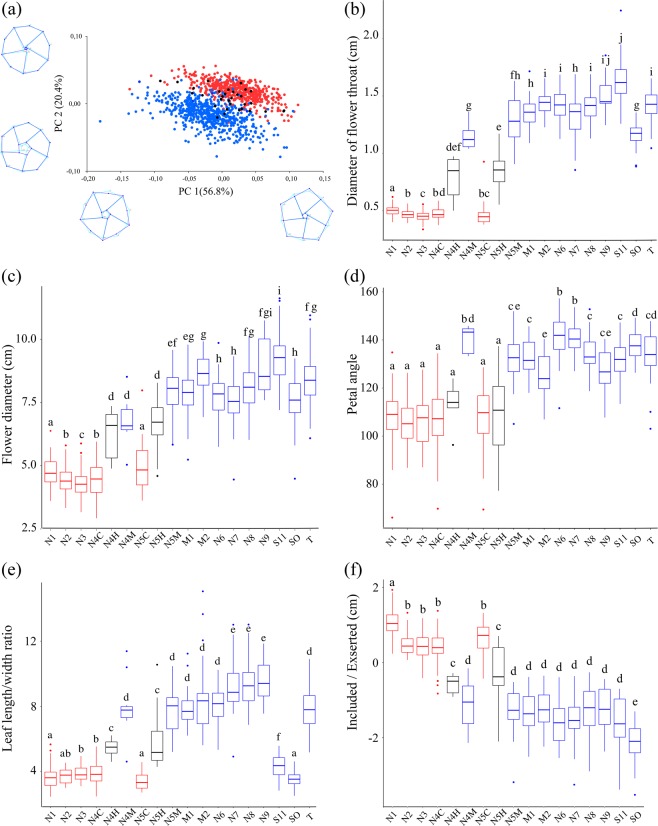
Flower trait analysis. (**a**) Principal component analysis of morphospace. Schematic flower outlines point 15 anatomical landmarks (dots) and measured parameters (lines). Panels (**b**) to (**f**) represent descriptive statistics of the canga-delimited trait diversification illustrated with boxplots. The band inside the box is a median value; box spans the upper and lower quartiles; box-connected lines are whiskers showing the lowest and highest datum still within 1.5 interquartile range; dots are outliers. Letters above each plot represent statistically significant differences at p < 0.05 according to the Wilcoxon posthoc test. Individual boxplots correspond to canga-delimited samples, the ID’s of which are listed along X-axis as cangas N1 to N9; S11 is S11 plateau canga; SO – Sossego granitic inselberg; T – canga Tarzan. Cangas N4 and N5 are sympatric and host putative hybrids N4H, N5H; *I. cavalcantei*, N4C, N5C; and *I. marabaensis*, N4M, N5M. Labels M1 and M2 correspond to the samples from cangas Morro 1 and Morro 2. Sample species identity is shown by boxplot colouring: red - *I. cavalcantei*; blue - *I. marabaensis*; black – putative hybrids that here are treated as an independent taxon. (**b**) Variation in flower throat diameter. (**c**) Flower diameter, when viewed along the tubular flower axis. (**d**) The angle between the tube axis and limb plane. (**e**) Leaf variation in shape and size among *I. marabaensis* populations exceed variation in *I. cavalcantei*. (**f**) Distances from the stigma to the plane at flower throat. Values >0 or <0 correspond to exserted or included styles, respectively.

To determine whether the flower shape diversification fits contrasting hummingbird versus bee pollination syndrome trait suites^38,39,45^, principal component analysis of morphospace defined by 15 anatomical landmarks on frontal flower images was carried out. PC analysis revealed two main components explaining 77.2% of phenotypic variation between *I. cavalcantei* (n = 507) and *I. marabaensis* (n = 647). The PC1 (56.8%) was related to a difference in limb contours. Sampled individuals of *I. cavalcantei* and *I. marabaensis* were grouped into distinct clusters that were separated by the PC2 (Fig. 3a; Supplementary Table S2). The PC2 (20.4%) emphasized on differences in flower throat aperture, narrower in *I. cavalcantei* and wider in *I. marabaensis*, which is consistent with an idea of specialization toward hummingbird pollination in *I. cavalcantei*.

### Flower and leaf shape traits distinguished canga-delimited populations and emphasized on both additive and dominant genetic effects in putative hybrids

To assess the combined effects of genetic drift and gene flow in shaping phenotypic variation, we quantified trait variation between canga-delimited populations using descriptive statistics of four flower and one leaf shape parameters. Results pointed out several emerging patterns (Figs. 3 and S5; Supplementary Tables S2, S3). Firstly, the statistically significant differences suggested a consistent subdivision of samples into groups. For example, throat width in tubular flowers can be a significant evolutionary trait that may control access to the nectar by size discrimination between flower visitors. Pairwise comparison of throat width between five *I. cavalcantei* samples indicated four distinct groups of populations (Fig. 3b). Secondly, the sympatry effect was noted. For the trait “Flower diameter”, *I. marabaensis* sample from sympatric canga N4 was different from all other conspecific samples, closer to *I. cavalcantei* samples and indistinguishable from the PH individuals (Fig. 3c). Thirdly, the analysis of leaf length/width ratio illustrated the distance effects, which showed that among Northern cangas, *I. marabaensis* individuals from N8 and N9 had the narrowest leaves in the species. The leaf width increased in allopatric cangas Morro 1, Morro 2, N7 and N6, the latter being closest (2 km) to sympatric canga N4 (Fig. 3e).

The value distributions in PH samples from N4 and N5 were pairwise similar, indicating a single group for all analysed traits. The mean values for three measured flower parameters were intermediate between *I. cavalcantei* and *I. marabaensis* samples from sympatric cangas, which is consistent with additive gene action (Fig. 3, Supplementary Table S3). The angle between the tube axis and limb plane assigned all *I. cavalcantei* and PH samples to a single group that was different from four groups of all *I. marabaensis* samples (Fig. 3d). This result indicated stabilizing selection on the angle trait in *I. cavalcantei* and genetic dominance.

### Variation in reproductive organ positions

To compare the role of reproductive organ positions, which are important adaptive flower geometry traits influencing the efficiency of pollen removal and deposition by distinct pollinators^45,70^, we analysed dissected flowers. In *I. cavalcantei*, five anther-bearing filaments, the so-called stamens, were similar in length and largely elevated over the flower throat, i.e., were exserted (Fig. 2g,h,l). Five stamens of *I. marabaensis* were included and differed in length, two longer than other three (Fig. 2k,n), indicating sub-functionalization, e.g., the long and short anthers in *Brassica rapa* made different contributions to pollination efficiency of nectar‐feeding bumblebees^71^. PH plants had five stamens of similar length, a phenotype that can be the second example of genetic dominance (Fig. 2i,m). Stigma-bearing styles (n = 358) were largely exserted in *I. cavalcantei* (Figs. 2l and 3f), which is consistent with adaptation to hummingbird-pollination. However, 24 styles were included (7%); 8, 12 and 4 were from allopatric N3 (9%, n = 90), sympatric N4 (14%, n = 82) and N5 (13%, n = 30), respectively (Fig. 2l). No style inclusion was found in N1 (n = 92) and N2 (n = 42). The styles were more exserted in N1 (1,1 ± 0.33 cm) as compared to four conspecific samples, i.e., 0.45 ± 0.37 cm (Fig. 3f). Samples of *I. marabaensis* (n = 354) were similar to each other, except Sossego population, and all had included styles (Figs. 2n and 3f).

In hermaphroditic flowers, spatial separation of the anthers and stigma, the so-called herkogamy, is a common strategy to reduce sexual interference between the respective male and female flower functions^72,73^. Measurements of the distance from stigma to the distal tip of the longest stamen in flowers showed that anthers were mostly positioned below stigmas, i.e., largely descending herkogamy, both in *I. cavalcantei* (90%) and *I. marabaensis* (82%) (Supplementary Fig. S5; Table S3). Nevertheless, flowers in which stigmas were in contact with anthers, or were below anthers can be found within variation range in both species (Fig. 2h,l,n). Herkogamy^72,73^ is considered an adaptive trait in self-compatible species, decreasing the likelihood of self-pollination and increasing the opportunity for outcrossing by abiotic or biotic pollen vectors. The role of herkogamy in plant-pollinator interactions of self-incompatible species is less clear^74^.

### *I. cavalcantei* and *I. marabaensis* are self-incompatible species

*Ipomoea* species can be either self-compatible or self-incompatible^74,75^. To understand the reproductive mode, we isolated flowering shoots of *I. cavalcantei* (canga N1, n = 16; N4, n = 11) and *I. marabaensis* (N6, n = 15; N8, n = 15). Individuals produced 603 and 557 flowers, respectively, none of which developed into fruit capsules (Supplementary Table S4). In *ex-situ* collection, hand-pollination of flower pistils with pollen from the same flower, i.e., self-pollination did not produce seeds both in *I. cavalcantei* (27 flowers of 10 individuals) and *I. marabaensis* (14 flowers of 3 individuals) (Supplementary Table S5). Analysis of natural progeny from six *I. cavalcantei* mothers in sympatric canga N4 showed outcrossing (Supplementary Table S6). Next, we performed intraspecific crosses (Supplementary Table S7). Out of nineteen tested *I. cavalcantei* parental combinations, the 82% seed set was obtained from only ten plant pairs. Similar cross-pollinations between *I. marabaensis* plants gave 85% seed set from four combinations out of seven tested. Genotype-dependent rejection of pollen deposited on stigmas is characteristic of gametophytic or sporophytic self-incompatibilities in plants and can explain the seed set failure in some parental combinations. Thus, four lines of experimental evidence suggested self-incompatibility (SI) genetic system in both species, implying an obligatory dependence of *I. cavalcantei* and *I. marabaensis* reproductive success on biotic and abiotic pollen vectors.

### Homoeologous single nucleotide polymorphisms were infrequent

To identify molecular markers that can be used for species identification, the homoeologous single nucleotide polymorphisms (h-SNP)^76^, we determined partial sequences of ten nuclear genes of *I. cavalcantei* (n = 46), *I. marabaensis* (n = 48) and PH plants (N4, n = 5; N5, n = 20). In a total of 6097 base pairs (bp), 128 and 135 single nucleotide polymorphic sites (SNP) characterized *I. cavalcantei* and *I. marabaensis*, respectively (Supplementary Table S8). Among 161 SNP’s found in both species, five were multiallelic (one in *ANS*, one in *WD40* and three in *UF3GT*), others were biallelic. In our sampling, likely h-SNPs occurred only in *MYB* and *F3*′*H* (Fig. S6). At other polymorphic sites, identical bases were found in both species, which indicated that the majority (97%) of found SNPs reveal molecular diversification within the species, supporting the idea of a close interspecific relationship.

### Interspecific genetic differentiation supported the known functional role of *F3*′*H* and *MYB* gene loci in flower colour shift from blue to red

**Figure 4:**
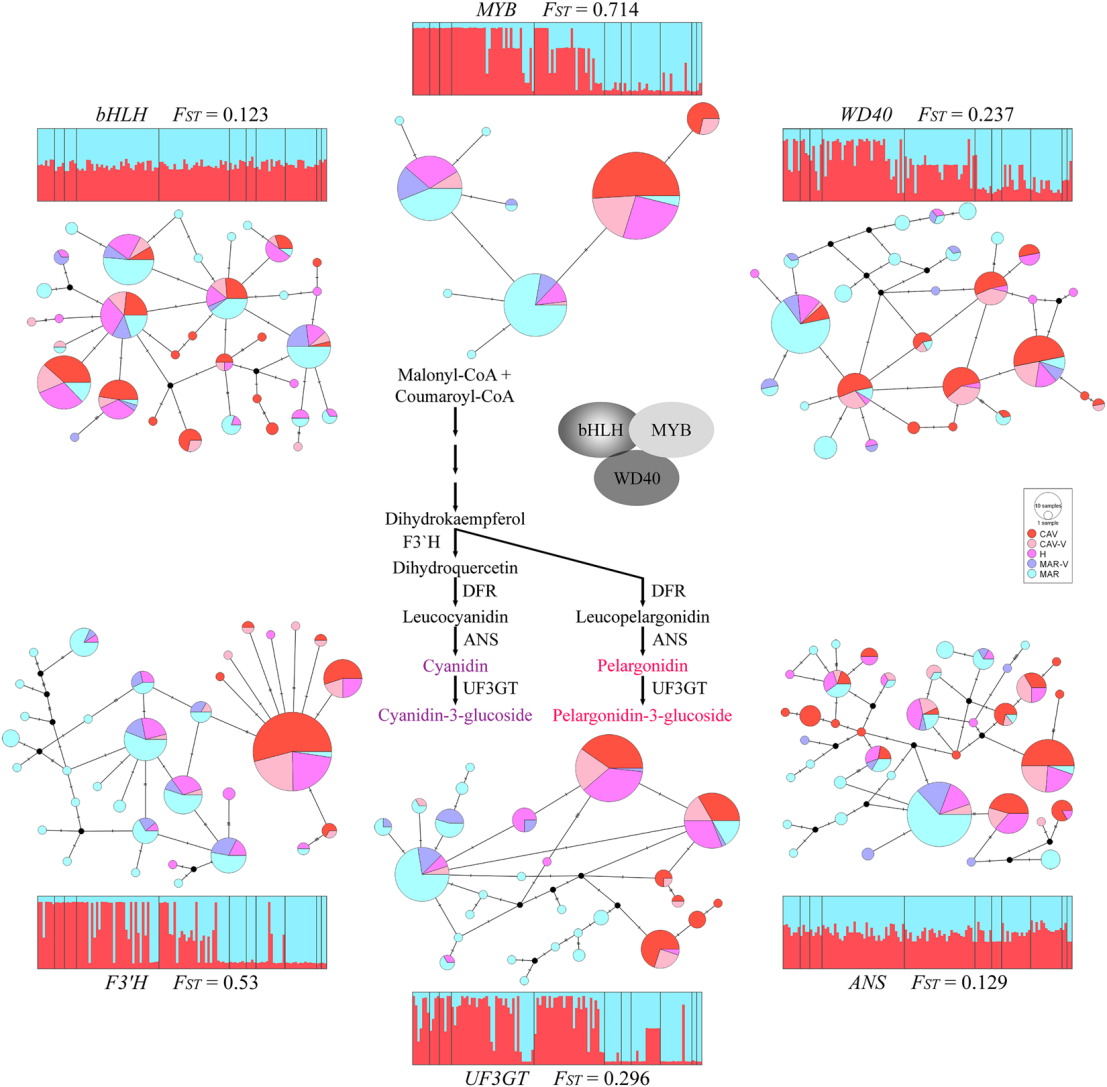
Anthocyanin biosynthesis pathway. Schematic showing the pathway to anthocyanin pigments coloured red or purple. Flavonoid 3′ hydroxylase, F3′H; anthocyanidin synthase, ANS; UDP-glucose:flavonoid 3-O-glucosyltransferase, UF3GT. Gene for dihydroflavonol 4-reductase, DFR was not analysed. Pathway regulator is a transcription complex MYB-bHLH-WD40. The haplotype networks, single locus multiallelic STRUCTURE plots (K = 2) and interspecies genetic differentiation (*F*_*ST*_) calculated for individual genes are shown. Individuals in STRUCTURE plots are ordered as in Fig. 5.

To characterize gene allele diversity and distribution, we phased polymorphisms into haplotypes, which showed that each gene was represented by a minimum of 4 to a maximum of 25 alleles in *I. cavalcantei MYB* and *I. marabaensis ANS*, respectively (Supplementary Table S8). The near-complete interspecific separation among major tested haplotypes was evident only for the *F3*′*H* and *MYB* gene orthologs (Figs. 4 and S6; S7). Major haplotypes in other genes were commonly shared between the species. For example, *bHLH* haplotypes H01, H02, H03, H04, H05, H08, and H15, which account for 167 chromosomes out of the 228 scored (73%), were all found between both *I. cavalcantei* and *I. marabaensis* individuals. The network haplotype partitioning agreed with standard population genetics statistics. The pairwise *F*_*ST*_ varied from 0.07 among *RPB2–2* haplotypes to 0.53 and 0.71 amid *F3*′*H* and *MYB* haplotypes, respectively (Supplementary Table S8). Thus, the identification of h-SNPs and high interspecies genetic structuring is consistent with a functional role of *F3*′*H* and *MYB* in a flower colour shift^64–66,68^. Analysis of dN/dS ratio’s^77^ in our dataset of partial protein coding regions suggested only stabilizing selection and indicated intragenic recombination in *WD40* (Supplementary Table S9).

### Interspecific gene admixture analysis suggested fertility of F1 natural hybrids

**Figure 5:**
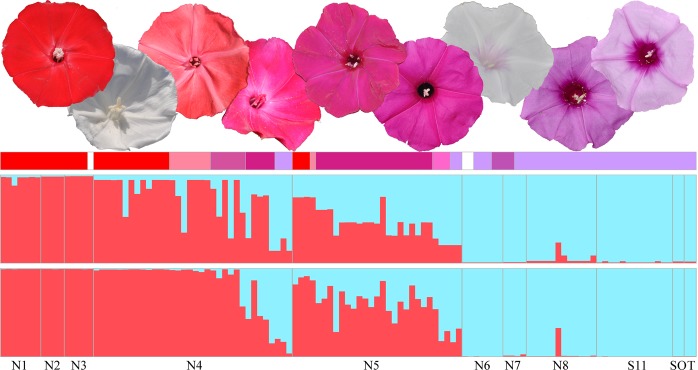
Genetic admixture. Two STRUCTURE plots (K = 2) are shown. The upper plot was build using multiple alleles at two loci, *F3*′*H* and *MYB*. Ten multiallelic loci were analysed in the lower plot. Individuals are ordered by locations, i.e., cangas of origin. The bar above STRUCTURE plots indicates the flower colour of analysed individuals, red for *I. cavalcantei* and lavender for *I. marabaensis*. Flower colours of other analysed individuals were white, pink, purplish, magenta, and intense lavender.

To characterize interspecific hybridization molecularly, we analysed ten multiallelic loci using software STRUCTURE^78,79^ that allows distinguishing F1 hybrids from their progeny and detect introgression between species in animals and plants^80–83^ (Fig. 5). The admixture coefficient Q values between 0.4 and 0.6 suggested ten F1 interspecific hybrids in our dataset, of which nine had magenta flower colour (Supplementary Table S10). Potential seventeen F2 or backcross (BC) individuals with Q values between 0.6–0.9 (twelve samples) and 0.1–0.4 (five samples) indicated higher rates of backcrossing to *I. cavalcantei*. The Q cut-off 0.1–0.4 suggested admixture by introgression in three *I. marabaensis* plants: a pink flower plant from N5 and lavender flower plants from N4 and N8. *I. cavalcantei* plant with purplish flower colour (Q = 0.884) can be an admixed individual. The observed distribution of h-SNPs can be due to introgression or incomplete sorting (Fig. S6). In introgression scenario, three *I. marabaensis* in S11 and one in N4 were heterozygous carriers of *I. cavalcantei-*specific *MYB* h-SNPs; two plants in N8 and one in N4 were heterozygous carriers of *I. cavalcantei-*specific *F3*′*H* loci h-SNPs; four flower colour variants of *I. cavalcantei* carried *MYB* h-SNPs of *I. marabaensis*. Thus, molecular marker admixture analysis suggested that natural interspecific hybrids can be fertile, which is a necessary condition for gene flow.

### Magenta flower hybrids comprised self-incompatible, male and female fertile individuals

To validate the molecular admixture analysis results, first, we followed the seed set by the plants with magenta flowers in canga. Most hybrid plants produced seeds, showing the natural generation of F2 or/and BC progeny. Seeds were viable and the resulting offspring seedlings developed into photoautotrophic plants maintained in growth chambers. We genotyped progeny of four plants, EB138 (Q = 0.71), EB047 (Q = 0.86), EB015 (Q = 0.56) and EB081 (Q = 0.44). All analysed offspring were a result of outcrossing (Supplementary Table S6). To verify whether this result can be explained by self-incompatibility or male sterility, we transferred four F1 hybrids to *ex-situ* collection (EB015, Q = 0.56; EB022, Q = 0.41; EB081, Q = 0.44; EB088, Q = 0.41) and tested them for seed set after assisted self-pollination. All self-pollinated flowers aborted (Supplementary Table S5). Pollen staining suggested 95–99% pollen viability (Supplementary Table S11), arguing against meiosis abnormalities. To determine whether viable pollen is functional, we pollinated flowers of several *I. cavalcantei* and *I. marabaensis* mother plants, which resulted in seed development in some parental combinations (Supplementary Table S12). The results strongly suggested functional SI in hybrids, which can explain the failure of crosses in some of the parental combinations. To find out if hybrids will accept pollen from either *I. cavalcantei* or *I. marabaensis*, reciprocal pollination were performed. Seeds developed with some, but again not all parental combinations regardless of the species pollen origins (Supplementary Table S12). We also obtained F2 progeny seeds from some crosses between hybrids, e.g., EB081 × EB015; EB081 × EB161 (Q = 0.62). Thus, four tested F1 hybrids are likely to be fully male and female fertile.

### Species cross-hybridized in real-time

To determine the potential directionality of interspecific hybridization that can be underpinned by the postmating reproductive barriers, events from pollen deposition on stigma to zygote formation, we performed crosses based on mother-father matrix comprising several individuals of *I. cavalcantei* and *I. marabaensis*. The outcome of both intraspecific and interspecific crosses was “either all or nothing,” depending on a given combination of individuals (Supplementary Table S13), which is consistent with the operation of homologous SI systems. Viable interspecific F1 hybrid seeds were produced irrespective of the cross direction. We obtained seeds also by pollinating *I. cavalcantei* rescued from sympatric canga N4 by pollen of *I. marabaensis* originating from remote allopatric canga N8 or granitic inselberg near copper mine Sossego. Comparable rates of seed set after hand-pollinations was observed in four directions (Supplementary Tables S7, S13). Thus, *I. cavalcantei* and *I. marabaensis* readily cross-hybridized by hand-pollination.

To determine whether interspecific hybridization can occur during a single growing season in nature, we collected seeds from a red-flowered *I. cavalcantei* migrant in canga N8 and a lavender-flowered *I. marabaensis* plant from sympatric N5 (Supplementary Fig. S2). Genotypes of progeny plants were compared to the genotypes of the mothers at *MYB* and *F3*′*H* gene loci by scoring h-SNPs. Three fruit capsules collected from a migrant gave eight seeds (maximal expected 12). All eight seeds were viable. Hundred percent of offspring plants were interspecific heterozygotes at two loci. Tested *I. marabaensis* mother grew near five red flowered *I. cavalcantei* and magenta flowered EB161 (Q = 0.62) and EB162 (Q = 0.7) hybrids. Nine collected fruit capsules gave 14 seeds (expected 36) of which 12 germinated. Three offspring plants were interspecific heterozygotes at *F3*′*H*, suggesting fertilization with pollen from either *I. cavalcantei* or hybrids. The *MYB* locus was not informative, because *I. marabaensis* was an interspecific heterozygote at this locus, possibly an admixed individual. Thus, *de novo* bidirectional formation of natural interspecific hybrids was ongoing in N8 and N5.

### The nectar rewards of *I. cavalcantei* and *I. marabaensis* flowers were similar and relatively large

To determine whether studied morning glories offered any rewards for visiting pollinators, in addition to pollen, we analysed nectar production. Flowers of *I. cavalcantei* plants (n = 3) and *I. marabaensis* (n = 3), produced 64 ± 19 μL and 75 ± 13 μL of nectar, respectively (Supplementary Table S14, S15). Four F1 hybrids produced on average 77 μL of nectar. As compared to six Argentinian *Ipomoea* species^84^ and 46 plant species from Brazil Cerrado savannahs ecosystems^85^, both morning glories from canga produced the largest volumes of nectar, suggesting a potential to reinforce and modulate pollinator behaviour through reward economics^45^.

### Flower visitor assemblies of native animal species overlapped

**Figure 6:**
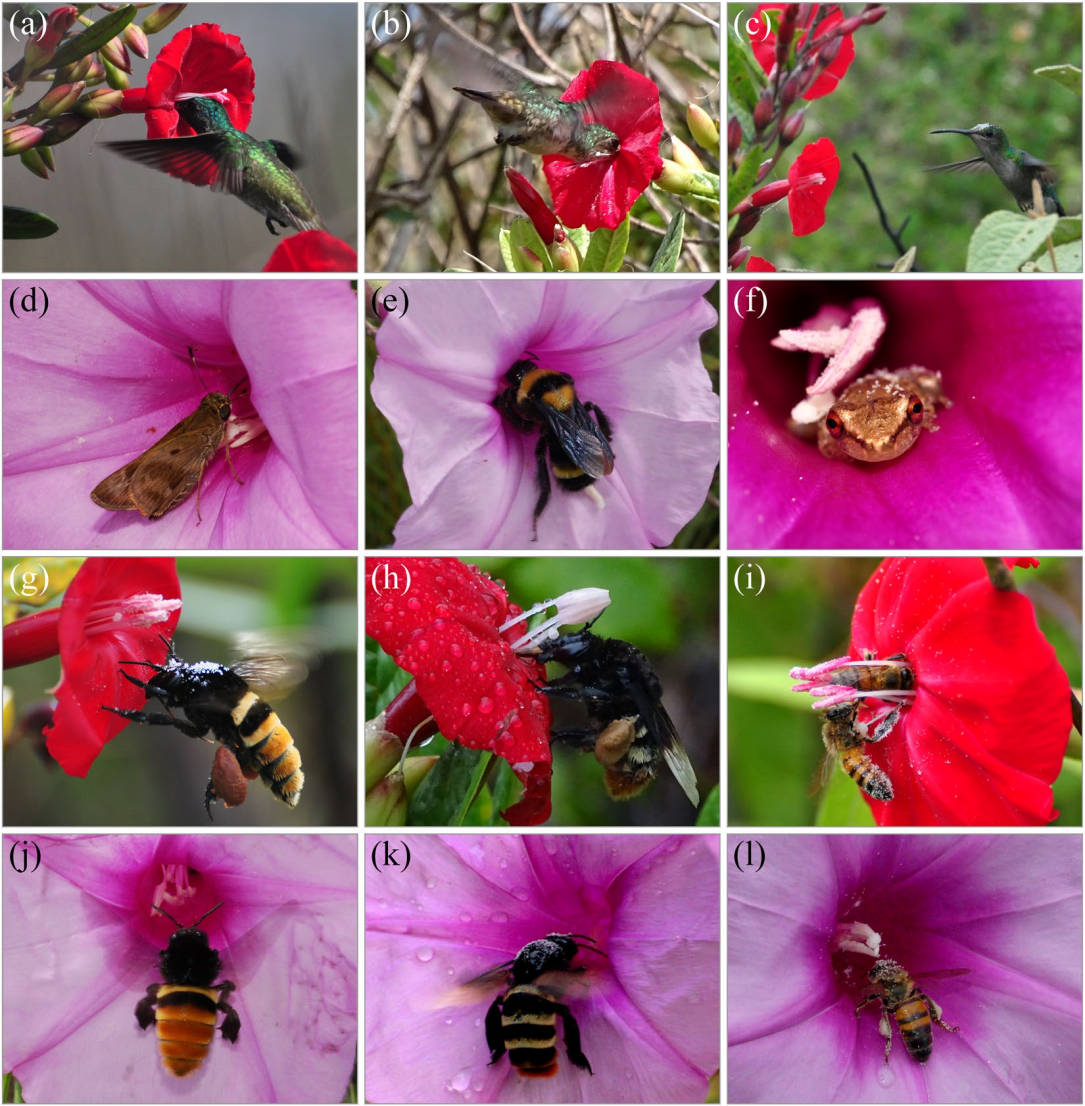
Flower visitors. Species-specific flower visitors as found in this study are in (**a–c**) for I. cavalcantei and in (**d–f**) *for I. marabaensis*. Species shared flower visitors are in (**g**–**l**). (**a**) Anthers and style are in close contact with hummingbird head. (**b**) Contact with bird throat. (**c**) Hummingbird with pollen on forehead approaches a flower. (**d**) Butterfly. (**e**) Bumblebee *Bombus transversalis*. (**f**) *Scinax* spp. tree frogs were legitimate flower visitors. Note pollen on the body. (**g,j**) Orchid bees *Eulaema cingulata*. In (**g**), pollen is shed on the animal thorax; the animal long tongue is visible. (**h,k**) Orchid bees *Eulaema bombiformis.* (**i,l**) *Apis mellifera* honeybees. In (**i**), one honeybee is just exiting flower tube, the body of the second animal contacts the flower stigma. All honeybees are covered in pollen.

To determine whether hummingbirds forage nectar on morning glories, we conducted field observations at allopatric N1, sympatric N4, N5, and allopatric N6. At least nine species of hummingbirds foraged nectar on *I. cavalcantei* (Figs. 6a–c; and S8). The birds also visited pink flowers of *I. cavalcantei* and magenta colour flowers. We did not see hummingbirds visiting *I. marabaensis* flowers in sympatric canga N4, N5 or in our *ex-situ* collection. To verify this observation, we compared hummingbird response to the flowering shoots of *I. marabaensis* and *Dyckia duckei* (family *Bromeliaceae*)^86^ in allopatric canga N6 where species grew in proximity to each other often as close as half a meter. Hummingbirds were commonly feeding on the orange flowers of the bromeliad but ignored *I. marabaensis* flowers during the ten days of 76-man-hours observations by two people. The results suggested active morning glory species discrimination by the hummingbirds at the study locations.

To identify other potential pollinators, we surveyed flower visitor communities of *I. cavalcantei* as compared to *I. marabaensis*. Orchid bees *Eulaema cingulata*, *E. bombiformis* foraged on flowers of both species (Fig. 6g,h,j,k). These bees have 20–25 mm long tongues^87^, which are likely to enable animal’s access to nectar in *Ipomoea* tubular flowers despite the large animal body size. Interestingly, pollinator switches from short-tongued bees via long-tongued bees to hummingbirds appear to have taken place repeatedly in the genera *Nasa*, *Loasa* and *Caiophora*^88^; nectar offerings are high in both orchid bee-pollinated species and hummingbird-pollinated species in a genus *Costus*^89^. *Trigona* spp. bees were frequent visitors of *I. cavalcantei*, *I. marabaensis*, and magenta flower hybrids flowers (Supplementary Fig. S9). *Trigona* bees can have a dual role as pollinators and nectar robbers in both plant species. Besides, *Trigona* bees often destroyed stamens and styles, reducing plant reproductive success (Supplementary Fig. S9). Other common nectar robbers were carpenter bees and some hummingbirds (Supplementary Fig. S9). The *I. marabaensis*-specific legitimate flower visitor community included butterflies, two species of bumblebees (*Bombus brevivillus*; *B. transversalis*) and *Scinax* spp. tree frogs (Fig. 6d–f). Interestingly, tree frogs were consistently found inside of *I. marabaensis* flowers on all days of fieldwork in canga N6. Various species of beetles were legitimate visitors, but species differed between morning glories. Thus, native bee species exemplified an overlap between flower visitor assemblies.

### Alien Africanized honeybees foraged on *I. cavalcantei* and *I. marabaensis* flowers

Africanized race of honeybees has been introduced to Brazil in 1957^90,91^. In canga, we observed *Apis mellifera* identified as an Africanized race (Supplementary Fig. S10). To determine if alien pollinator can have any role in our model, we characterized honeybee behaviour in greater detail. Honeybees foraged nectar and pollen on *I. marabaensis* (Fig. 6l) and were also visiting *I. cavalcantei* flowers (Fig. 6i; Supplementary Video 1) at a frequency one honeybee per plant every minute (Supplementary Table S16). Honeybees were persistent in attempts to enter *I. cavalcantei* flower tubes, even in cases when flowers have been already occupied either by another honeybee or the beetles or by *Trigona* bees (Supplementary Video 2). Honeybees had difficulties in exiting relatively narrow *I. cavalcantei* flower tubes (Supplementary Table S17; Supplementary Video 3). For 27% of the bees, it took longer than 30 seconds to exit the flower, while many bees were trapped for up to 8 hours (Supplementary Video 4). 68% of visiting honeybees did not touch *I. cavalcantei* stigmas (Supplementary Table S17). On some exits, bees were not ending on a flower limb but were entangled in a filament-anther-style bundle, which can promote inter-individual pollen transfer by honeybees (Supplementary Video 5), emphasizing on the interplay between flower geometry and pollen transfer by vectors. In canga N1, we found three dead honeybees inside of *I. cavalcantei* flowers, one in a flower tube filled with rainwater, indicating that flower tube exit difficulties can increase honeybee mortality while foraging on *I. cavalcantei* (Supplementary Video 6). We collected eighteen rainwater-free flowers with trapped honeybees; four honeybees were found dead the next day morning. Results suggested that honeybees were both attracted and maladapted to *I. cavalcantei* flowers, while for some animals it was a fatal attraction.

## Discussion

Here, we provide phenotypic and molecular evidence for the natural interspecific hybridization between taxonomically recognized red flowered *I. cavalcantei*^59^ and lavender flowered *I. marabaensis*^60^. We show that four magenta flowered plants with an admixture coefficient (Q) around 0.5 were fertile, bidirectionally crossing with parental species and in between. It was not feasible to assess in the wild the Bateson-Dobzhansky-Muller incompatibilities^92^. In the laboratory, all F1 and F2/BC natural and artificial offspring between *I. cavalcantei, I. marabaensis* and magenta flowered plants were fully autotrophic and did not show hybrid necrosis. Thus, available data indicated weak postzygotic isolation in our model, similar to the case studies in plant genera *Aquilegia*^93,94^, *Petunia*^95^, *Costus*^96^, *Caiophora*^88^, *Mimulus*^44^. In contrast, at least four examples of the incipient intraspecific postzygotic incompatibilities underpinned by different mechanisms distinguish ecotypes of *Arabidopsis thaliana*^9,19,20,22^. Multiple gene flow barriers characterize sunflower ecotypes radiating by adaptation to different habitats^97^. Sterility due to pollen unviability separates three species and some subspecies in a serpentine soil specialist *Streptanthus glandulosus* species complex^27^. It is not well understood why plant lineages vary in evolvement and establishment of postzygotic isolation^7,8^. The establishment of a complete reproductive isolation between lineages of fruit fly requires 4 My^98^. Time-to-speciation among plants and animals is estimated at 2 My^99^; 1 My in recent species radiation of African cichlid fish and Andean *Lupinus*^100^ but full reproductive isolation requires more than 10–14 My in interfertile tulip trees^101^, 16 My in largely interfertile species in the genus *Jamesbrittenia*^102^ and 60 My in ferns^103^. To understand whether divergence time insufficiency is one critical reason for weak postzygotic isolation and to reconfirm the taxonomic assertion of the sister species status^60^, it will be essential to estimate the age of *I. cavalcantei* and *I. marabaensis* diversification by the detailed phylogenetic reconstruction^100^ of the *Ipomoea* genus clade Murucoides^62^.

The described diversification in floral traits was consistent with contrasting specialization towards pollination by hummingbirds in *I. cavalcantei* versus bees in *I. marabaensis*. Keeping in mind that legitimate flower visitation by the animals does not identify the actual and the most efficient pollinators^45,104^, our field work suggested that hummingbirds and bumblebees could mediate reproductive floral isolation between *I. cavalcantei* and *I. marabaensis*. However, we also show that orchid bees, *Trigona* bees, and alien honeybees were frequent legitimate visitors of both species, potentially lowering the efficiency of floral isolation. The studied morning glories are perennials of unknown longevity in the wild. Magenta flowered hybrids could be decades old, indicating that interspecific hybridization is a rare event and floral isolation is rather stringent. The real-time interspecific hybridization in canga N8 and N5 showed both functionality of the overlap in flower visitor assemblages and substantial imperfection of floral isolation. This conclusion is in line with a consensus that floral isolation usually acts in concert with other prezygotic and postzygotic isolation barriers to gene flow^12^. Our controlled pollinations showed an efficient bidirectional interspecific hybridization between *I. cavalcantei* and *I. marabaensis*, suggesting weak postmating prezygotic isolation. In several plant species, floral isolation is enforced by ecogeographic isolation, such as adaptations to growth at different altitudes^8,9^ or to different soils^10,27^. In the Carajás mountain range covered by the rain forest, canga savannah-like ecosystems evolved on the sparsely distributes plateaus that are found at similar altitudinal elevations, and are separated by the maximum of 40 km to the distant S11, and by a maximum of 17 km in the Northern range^62^. *I. cavalcantei* and *I. marabaensis* are found in all types of canga microhabitats, such as shrubby patches, exposed iron-laterite rocks, wetlands, and grasslands^62^. Our previous common garden tests of growth substrates from different cangas did not reveal an effect on *I. cavalcantei* and *I. marabaensis* seedling growth^62^. Sympatry and migration sites characterized here can be considered as the natural reciprocal transplant experiments. Collectively, these findings do not support a critical role of habitat specialization as floral isolation reinforcement mechanism. Thus, current data point insular-type geographic isolation^100^ as a significant gene flow barrier between morning glories in canga savannahs. Plant species can arise in sympatry^11,105^, but most models of speciation by floral diversification^40,41^, gradual or snowball accumulation of postzygotic barriers^106^ assume some degree of the initial geographic isolation. Whether geographic isolation as a barrier to gene flow can be used in delineation of the taxa boundaries is questionable^8^ but it has been one of the major arguments in description of a new orangutan species^107^. Delineation of *I. cavalcantei* and *I. marabaensis* either as species, or subspecies, or morphotypes of the same species matters for the species-counting-dependent disciplines such as macroecology and conservation biology, and in communication with public and regulatory agencies^3,108,109^. The major eco-evolutionary question concerns the stability of *I. cavalcantei* and *I. marabaensis* floral diversification at the face of gene flow^110^. Genetic analysis of plant species with contrasting pollination syndromes showed tight genetic linkage of loci specifying major pollination syndrome traits^111,112^, such as visible colour, UV absorption, floral scent production, pistil length, and stamen length in the genus *Petunia*, leading to the idea of “speciation island” that resists dissolution by recombination in interspecies hybrids^111^. Pleiotropy, chromosomal inversions, tight genetic linkage and single allele mechanisms^110^, as well as divergent selection^113,114^ can counteract homogenizing effects of gene flow. Our screening of phenotypic variation provides initial cues about genetic linkage between flower traits. Genetically separable traits appear to be the colour hue and intensity, e.g., intense red versus pink; pistil insertion; the general morphologies of the flower and leaf. We did not observe obvious examples of uncoupling between the flower colour hue and flower tube morphology, with an exception of the pink coloured *I. marabaensis*-like flowers in canga N5. The relative rarity of the colour variants in *I. cavalcantei* is suggestive of divergent selection.

We show the generation of the interspecific natural F2/BC progeny; admixed individuals that largely resembled either of the parental species, which strongly argue for the interspecific gene flow in our model. Gene flow can explain the occurrence of *I. cavalcantei*-specific h-SNP’s in *I. marabaensis* populations from S11 and N8; however, this finding is inconsistent with our proposal of the key role of *MYB* and *F3*′*H* in flower colour diversification. It is possible that both *I. cavalcantei MYB* and *F3*′*H* gene alleles must be present in a plant to affect the flower colour, which was not a case in canga S11 and N8. Alternatively, genetic recombination could have separated critical regulatory *cis*-elements from the coding gene regions studied here, as in *Antirrhinum majus* model where recombination can separate downstream regulatory enhancer and coding regions in a gene with a key role in gene flow barrier^113^. The idea of the interspecific flow acting in concert with genetic drift is supported by the phenotypic analysis of flower and leaf traits. The presence of the flower colour intensity locus as in *Phlox drummondii* flower colour model^69^ can explain the pink, low intensity red, *I. cavalcantei* flowers and intensely coloured flowers in some *I. marabaensis* plants, for example. Introgressive adaptation, genetic, and demographic rescues are considered as beneficial consequences of interspecific hybridization for the species. From the ecological perspective, both ecosystems modeling^115^ and field studies^116,117^ demonstrated that intraspecific genetic variation could play a key role in structuring ecological plant-herbivore networks, which may, in turn, affect network persistence at a face of extrinsic and environmental ecological changes. We show intraspecific variation in geometry of plant reproductive organs, flower shape, size, which could impact the reproductive success of individuals, e.g., *I. cavalcantei* plants with inserted styles can benefit from pollination by the recently arrived honeybees. Plants can respond very fast, within a few generations, to a change in pollinators^118^. Thus, the current, probably stochastic, flower geometry and colour variation has a deterministic potential for adaptation to the changing environments and contrasting selective pressures imposed by the evolving pollinators and herbivores communities.

## Methods

### Canga ecosystems and study organisms

Amazon canga savannahs comprise 856 species of seed plants^61^. *Ipomoea cavalcantei*^59^ is only known from five cangas, N1 to N5^62,63^, that collectively measure about 20 km^2^. The closely related species *I. marabaensis*^60^ is more broadly distributed^63^, common in other canga and occurs on granitic inselberg near copper mine Sossego^62^. Cangas can be subdivided into microhabitats, such as grasslands, exposed iron lateritic rocks, shrubby vegetation, small wetlands^61^. We found studied morning glories in all homologous terrestrial microhabitats and along altitudinal gradients (lowest 656 m to highest 813 m, average 700 m above sea level) encountered in canga ecosystems^62^.

*I. cavalcantei* and *I. marabaensis* are perennial morning glories with woody stems and enlarged storage roots. In shrubby habitats, both species are vines that often reach the canopy (up to 6 meters) to display their flowers. In open habitats, plant stems are short and erect, i.e., shrubby habit. Flowers of both species last less than a day and were already fully open at five o’clock in the morning when it is still dark. Flower limbs of *I. marabaensis* are mechanically weak, floppy and easily damaged by heavy rain and strong wind; depending of a cloud cover, limbs begin to wilt at mid-day or early afternoon. In contrast, *I. cavalcantei* flowers are mechanically strong; flower limbs are rigid and persist in full turgor up to 15–16 pm, corolla abscission happens at some time after sunset or the next day; flowers tissues were under strong turgor to the extent that it was not unusual to see flower tubes burst open along the axis. Post- anthesis, flowers can shed at an abscission zone in the peduncle, most commonly if not fertilized, in both species.

### Screening for flower colour variation and establishment of *ex situ* collection

We assumed that most of the deviations in flower colour can be underpinned by low-frequency recessive alleles and that homozygous plants showing phenotype will be relatively rare. In the field screening, therefore, we intended to cover the maximal areas of cangas to identify colour variants, i.e., screening in all 13 cangas false coloured in Fig. 1a and in inselberg Sossego. To minimize the damage to the ecosystem in dense shrubby habitats and canga-forest boundaries, we visually scanned the vegetation canopy from the roads or paths laid down by geological surveys in preceding decades. The machete-assisted entries into those habitats were made to access the individual with unusual colour flowers. Representative wild types and colour variants from canga-mine boundaries were rescued to *ex situ* collection by excavating plant storage roots and replanting them in VALE Zoobotanical park (Parauapebas, Pará, Brazil).

### A randomized sampling of phenotypic variation

To characterize standing phenotypic variation, individuals growing at least 5 meters apart were sampled at several sites per canga (Supplementary Table S1). For flower size and shape measurements, a single flower per individual was collected; sepals, anthers, and styles were removed. Intact flower corollas or dissected flowers were photographed from the front and the side next to a ruler. Digital flower images were used for trait measurements. For the leaf length/width ratio, five leaves per individual were measured with a ruler for the leaf lamina length along midvein and maximal width. The sample sizes are summarized in Supplementary Table S2.

### Geometric morphometry analysis

To evaluate the variation of the shape of the limbs, a file with the “*.tps” extension was created with the help of the software tpsUtil v.1.40 (http://life.bio.sunysb.edu/morph/index.html)^119^, which serves as a database for storing Cartesian coordinates of the anatomical landmarks highlighted in the images. Fifteen anatomical landmarks were manually marked using the software tpsDig v.2.12. The *.tps file was used as input in the software MorphoJ v.2.0^120^, where Procrustes fit was calculated. From the obtained values, a covariance matrix was calculated, which was used to calculate a principal components analysis in order to visualize the variation of form among the studied groups. The scatterplots of the first two main components were used to observe variation of the shape of the species in the morphospace. The effects of allometry were calculated through multivariate regression analysis^121^, procrustes residues being the dependent variables, and the size of the centroid, the independent variable^122^. A permutation test (10,000 cycles) was performed concomitantly with multivariate regression analysis as a test of significance^123^. The sample sizes are summarized in Supplementary Table S2.

### Trait descriptive statistics

The corolla diameter and the diameter of the tube opening were measured in frontal view, and in lateral view the length of the corolla tube and the angle formed by the petals using software ImageJ v.1.52a (https://imagej.nih.gov/ij/docs/guide/146.html)^122,124^. The tube length of the flowers was measured from the base of the tube to the opening of the petals. The angle was measured between the tube and the floral limb. To characterize reproductive organ geometry, distances from the stigma to the distal tip of the longest stamen (herkogamy) and from stigma to the plane of the flower throat were measured. For each linear and angle measurement, a Shapiro-Wilk test was performed to test the normality of the distribution. A Kruskal-Wallis test was performed to verify if there was a difference between the means of measurements between flowers from different cangas, with Wilcoxon post-hoc test. All statistical analyses were performed with software R v.3.4.1^125^. The graphs were generated with the help of the package ggplot2^126^.

### Reproductive mode, controlled pollinations, and seed viability

To understand whether species can produce seeds after self-pollination in the wild, we removed flowers at anthesis and developing fruits from the flower-bearing shoots, leaving at least ten flowers buds. Shoots were bagged with a nylon mosquito-proof mesh. Bags were tied at the bottom around the stems. Two months later, the bags were collected to count abscised flowers and to assess fruit set. The reproductive mode of the species and cross-fertility of individuals was also studied using controlled pollinations. All mothers in controlled pollinations were from *ex situ* collection. Since plants were maintained outdoors, flowers had to be isolated with cheesecloth bags. Flower buds at 1–2 days before anthesis were emasculated, covered by a bag which was sealed with a tread around flower peduncle. At anthesis, bags were cut-open at the top, pollen was deposited on stigmas using ethanol-sterilized forceps, and bags re-sealed. Pollen donors were either from *ex situ* collection or from canga residents. To avoid pollen contamination, anthers were taken only from either bag-isolated flowers, or from flowers that developed from harvested flower buds one to two days before anthesis; the latter kept on the water in an insect-free herbarium room of VALE Zoobotanical park. Hand-pollinations were performed between 7 am and 12 pm. The stigma receptivity window was not studied here. Pollination events were considered as failed when bagged flowers abscised within a week. Pollen viability staining^127^ was performed using anthers from flower buds one day before anthesis. Seed viability was tested by germination and seedling growth under controlled environment in growth chambers. A seed was considered as viable only if germinated seedling developed more than three fully expanded true leaves, i.e., was fully photoautotrophic.

### Flower visitors

Approximately 200 man-hours of field studies were dedicated for observations, still and digital video recordings of flower visitor communities, both illegitimate and legitimate, in cangas N6 and N8 (allopatric *I. marabaensis* populations); N1 (allopatric *I. cavalcantei*) and N4, N5 (sympatric canga). We collected alien honeybees for the race identification, which was carried out as detailed in Supplementary Fig. S10. We did not engage in field work at dawn and late night, thus nocturnal pollinator groups such as nectarivorous bats and species-rich moth were not followed.

### DNA extraction, PCR amplifications and amplicon sequencing

For gene allele frequencies analyses, the sampling of *I. cavalcantei* populations (total n = 46) included: (i) randomly selected red-flowered individuals (N1, n = 7; N2, n = 4; N3, n = 4; N4, n = 13; N5, n = 4); (ii) alternative colour selected plants, i.e. pink (N4, n = 7); purplish (N4, n = 6); white (N3, n = 1). For *I. marabaensis* (total n = 48), randomly selected samples included (N4, n = 3; N5, n = 2; N6, n = 3; N7, n = 2; N8, n = 12; S11, n = 13; SO, n = 2; T, n = 2); colour variants, i.e. intense lavender or purple (N6, n = 2; N7, N = 2), white limb (N6, n = 2), intense pink (N5, n = 3). The putative hybrid sample (total n = 25) comprised DNA from sympatric N4 (n = 5, all magenta coloured flowers) and N5 (n = 20, variable colours as in Fig. 1b). Additional DNA samples were sequences to analyse progeny plants for reproductive mode. DNA sequence dataset was prepared as previously described^62^. Primer sequences and modifications to PCR cycling programming^62^ are in Supplementary Table S18.

### Analysis of molecular variation

To analyse population genetic structure and gene flow between the species, we sequenced parts of 10 nuclear genes. Because of the blue-to-red flower colour shift, six genes in our dataset were likely orthologs of the *Ipomoea* Anthocyanin Biosynthetic Pathway (ABP) genes genetically and biochemically characterized in Japanese morning glory *I. nil*, common morning glory *I. purpurea* and cypress vine morning glory *I. quamoclit*. Three genes of ABP enzymes were: anthocyanidin synthase, ANS^68^; flavonoid 3′ hydroxylase, F3′H^64,128^ and UDP-glucose:flavonoid 3-O-glucosyltransferase, UF3GT^129^. One of the ABP regulators is a three-subunit MYB–bHLH–WD40 protein transcriptional complex that is known to be involved in a tissue-specific co-regulation of ANS, F3′H and UF3GT^130^, also in *Ipomoea*^131^. Therefore, we included orthologs of MYB1, WD40 and bHLH^132^. Next, we have chosen to analyse three genes for the twelve-subunit RNA polymerase II that is at the central core of eukaryotic genomes transcription^133^. In the *Ipomoea* genus, the RNA Polymerase II subunit 2 (*RPB2*) gene is duplicated^133,134^. Besides divergence in the encoded sequences of amino acid residues, the major difference between the *RPB2–1* and *RPB2–2* duplicated genes is a reduction in a number of introns in *RPB2–2*. This structural feature enabled the design of *RPB2–1/2* gene-specific primer pairs, avoiding gel purification of amplicons produced with previously recommended primers^133^. The third RNA polymerase II gene analysed here was expected to encode for subunit 3 (*RPB3*)^134^. The tenth gene was granule-bound starch synthase (*WAXY*) that plays a housekeeping role in carbohydrate metabolism and has been a popular gene in phylogenetic studies of *Ipomoea*^135^. Generated amplicons were end-sequenced and analysed by the online blast-N and blast-X tools at NCBI database^136^. We considered 93–99% DNA sequence identity over the entire length of the query as reasonable support for the gene orthology.

To identify polymorphic sites in our DNA sampling, nucleotide sequence chromatograms were aligned to a reference sequence using “map to reference” tool in the software suite Geneious v.11.0.3 (Biomatters). A high-quality sequence from *I. cavalcantei* or *I. marabaensis* was arbitrary chosen as a reference. Sequence mismatches and ambiguities were scored in excel files as polymorphic sites. To build haplotype networks, we used the software suite PopArt v.1.7^137^. Networks shown were built with the TCS algorithms as implemented in PopArt. The full-length haplotype sequences were used as well in calculations of population genetics statistics (observed and expected heterozygosity; nucleotide diversity π; Tajima’s D; Fu’s F_s_; *F*_*ST*_) with the software Arlequin v.3.5.2.2^138^. Genetic partitioning of individuals into groups/populations without any prior assumptions was performed with the software STRUCTURE v.2.3.4^78,79^. The data files for STRUCTURE analysis were either multilocus biallelic or multilocus multiallelic. In biallelic loci analyses, by default, we have chosen SNP’s that best discriminated well between *I. cavalcantei* and *I. marabaensis*. Twenty replicate runs were conducted for every value of K between 1 and 5, with a burn-in of 50.000 Markov Chain Monte Carlo (MCMC) steps followed by 50 000 iterations. The STRUCTURE run output files were further processed online by Structure Harvester vA.2^139^ and CLUMPAK^140^. The admixture coefficient (Q-value) generated from STRUCTURE was used to classify individuals into wild types and hybrids, using conventions proposed by others^80–83^. Plants were considered F1 hybrids when Q values ranged between 0.4 and 0.6. Plants with Q-values less than 0.1 or more than 0.9 were classified as wild type *I. marabaensis* or *I. cavalcantei*, respectively. Samples with Q values in ranges 0.1–0.4 and 0.6–0.9 were classified as backcrosses or F2 progeny^82,83^.

To comprehend evolutionary forces that have acted on genes, we analysed sequence alignments using web application Datamonkey 2.0^77^. Specifically, Fixed Effects Likelihood (FEL)^77^ maximum likelihood method was used to identify of sites that may have experienced pervasive diversifying or purifying selection by individually testing whether or not the ratio of relative rates of synonymous and nonsynonymous substitutions, dN/dS ≠ 1 at each site in the alignment; Mixed Effects Model of Evolution (MEME)^141^ performed a likelihood ratio test for detecting individual sites subject to episodic diversifying selection; positive selection affecting individual branches was estimated using the adaptive Branch-Site Random Effects Likelihood method (aBSREL)^142^, gene-wide episodic selection was estimated by Branch-Site Unrestricted Statistical Test for Episodic Diversification (BUSTED)^143^. In addition, screening alignments for evidence of phylogenetic incongruence, which can be a hallmark of recombination or gene conversion was performed using the software Genetic Algorithm for Recombination Detection (GARD)^144^.

### Parentage analysis

Seeds collected from individual mother plants were germinated and resulting progeny was grown in growth chambers. DNA was extracted from preserved leaf tissues of mothers and fresh leaves from progeny (PR) plants. Genotypes were identified by sequencing amplicons of four genes (*ANS, MYB, UFGT, WD40*). The parentage of offspring, i.e., self-fertilization or outcross, was inferred from genotyping at all four loci per individual. The progeny plant was considered as the result of pollination from a different individual than a mother only when the plant was scored as outcross at least at one locus. To assess real-time interspecies hybridization among migrants or at sympatry, h-SNP’s at *MYB1* and *F3*′*H* were scored in respective amplicons.

## Supplementary information

SUPPLEMENTARY INFORMATION

Easy exits by the honeybees

Persistence of honeybees foraging nectar on I. cavalcantei

Difficult exits

Trapped bees

Entangled honeybees

Trapped honeybees and the rain

## Data Availability

Data supporting the findings in this study are available from the corresponding author upon a request.
