# Supplementary material for: Geography is essential for reproductive isolation between florally diversified morning glory species from Amazon canga savannahs
Source: Sci Rep. 2019 Dec 2;9:18052. doi: 10.1038/s41598-019-53853-4 (PMC6889514; doi:10.1038/s41598-019-53853-4)
Supplement: Supplementary file 1 — SUPPLEMENTARY INFORMATION [file 41598_2019_53853_MOESM1_ESM.pdf]

SUPPLEMENTARY INFORMATION

Geography is essential for reproductive isolation between florally diversified morning glory species from Amazon canga savannahs.

Elena Babiychuk[1,6]*, Juliana Galaschi Teixeira[1], Lourival Tyski[3], José Tasso Felix Guimaraes[1], Luiza Araújo Romeiro[1], Edilson Freitas da Silva[2], Jorge Filipe dos Santos[1], Santelmo Vasconcelos[1], Delmo Fonseca da Silva[3], Alexandre Castilho[4], José Oswaldo Siqueira[1], Vera Lucia Imperatriz Fonseca[1] and Sergei Kushnir[5,6,7]

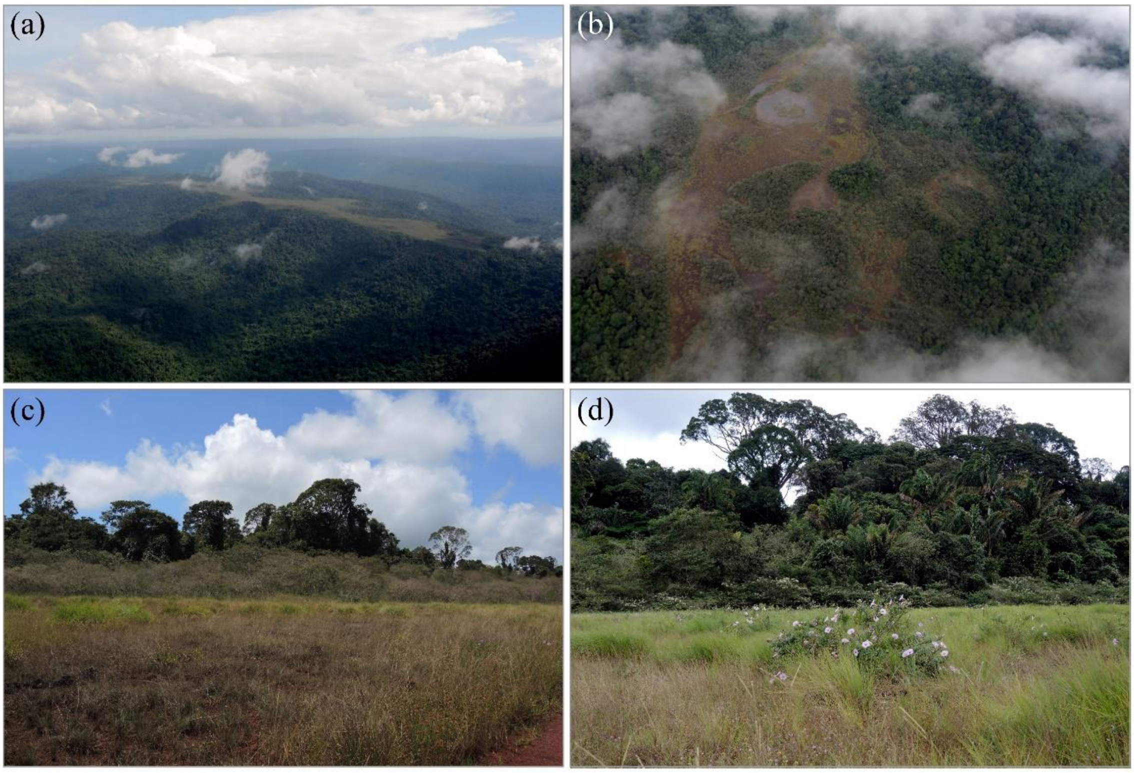

Supplementary Figure S1. Geographic barriers between cangas.

(a), **(b)** Aerial views of cangas N8 **(a)** and N7 **(b)** from a low flying airplane. Cangas are a light-green-brownish islands on a top of the mountains. Dark green is the mountain rain forest. Cangas are separated by the ravines between ranges.

(c), (d) Canga-forest boundaries. Cangas are bound by the rain forest, the canopy of which raises to 10-20 meters high. Allopatric N7 **(c)** at the beginning of dry season. Rainforest has few deciduous trees and remain in foliage around the year. Canga N6 **(d)** is populated by *I. marabaensis* growing here in a grassland as shrubs with erect stems.

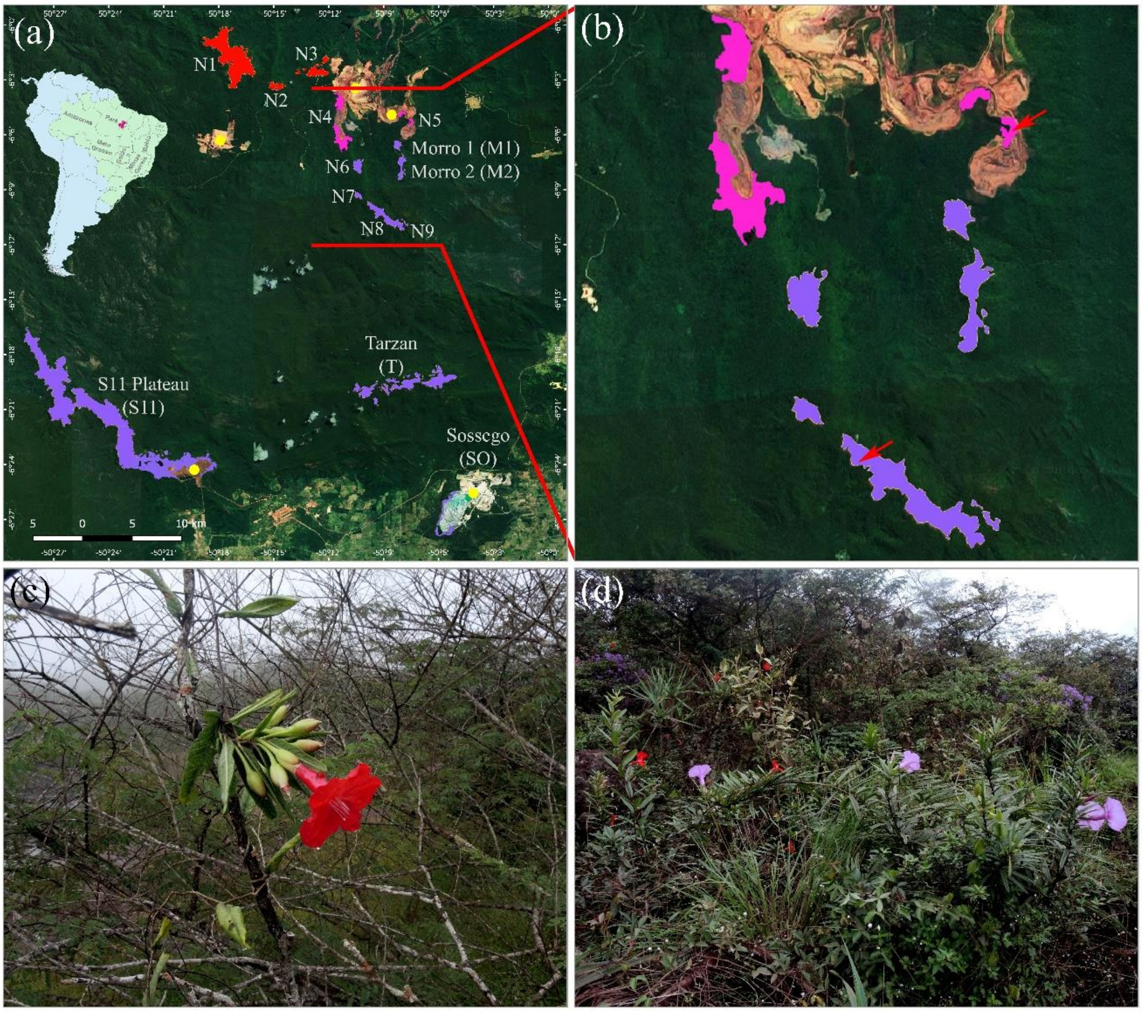

Supplementary Figure S2. Sympatry and migration.

(a), (b) Close up **(b)** of the Northern range of cangas found in Carajás National Forest **(a)**. Red arrows point sites where images in **(c)** and **(d)** were acquired. The shortest distance between sympatric canga N4 and allopatric N8 is 6 km. The labelling in **(a)** is as in Fig. 1a. The geographic map was generated with the software QGIS version 2.18 (http://qgis.org) based on satellite imagery source (https://mt1.google.com/vt/lyrs%3Ds%26x%3D%7Bx%7D%26y%3D%7By%7D%26z%3D%7Bz%7D&zmax=20&zmin=0) from Google (Google Maps satellite Carajás, Pará, Brazil; retrieved December 16, 2018). The map image was processed in Adobe Photoshop CS6 to indicate plant species ranges.

(c) *I. cavalcantei* migrant in canga N8.

(d) *I. cavalcantei* (six red flowers) and *I. marabaensis* (four lavender flowers) growing in sympatric N5.

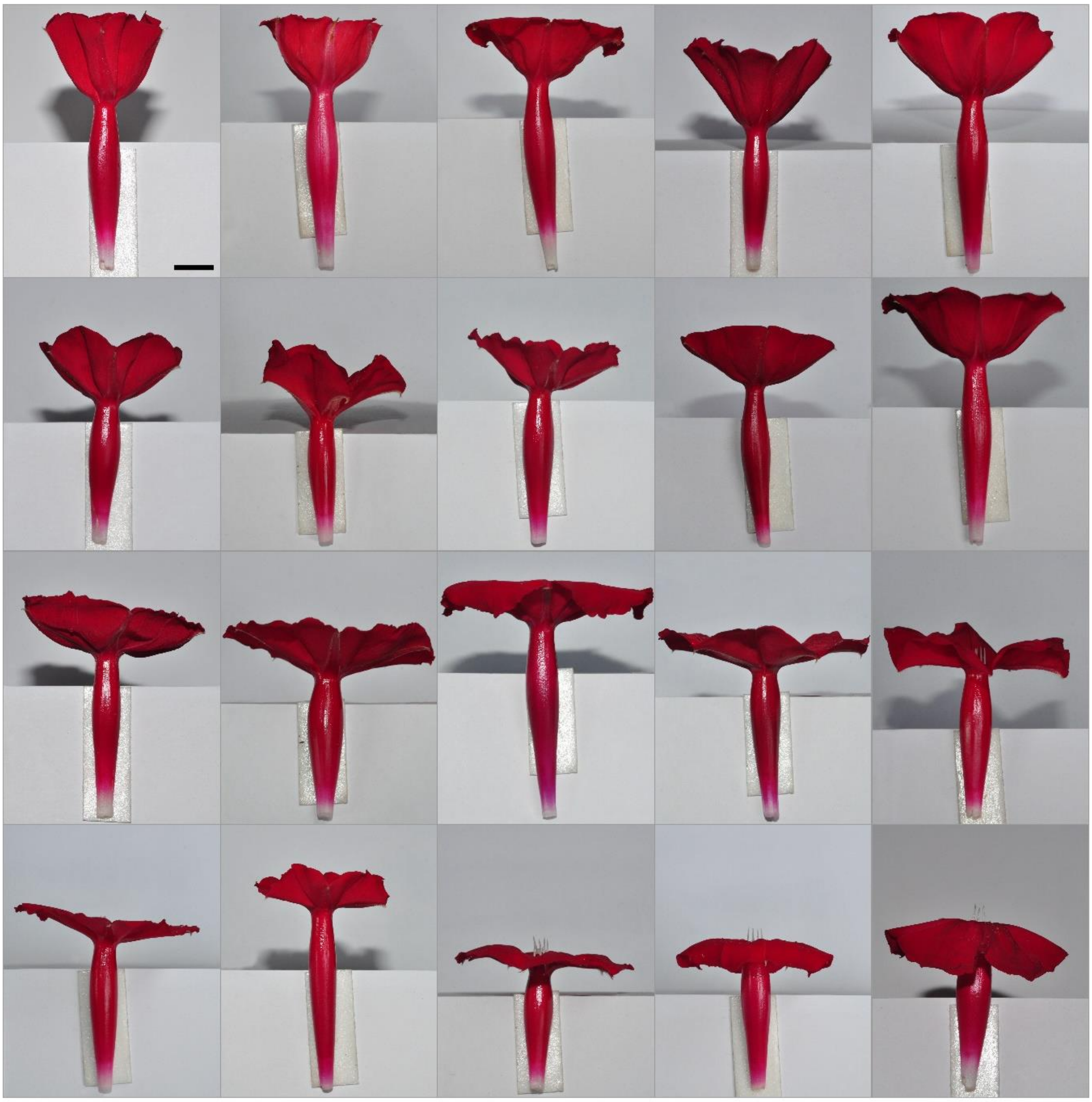

Supplementary Figure S3. Flower shape variation in *I. cavalcantei.*

For comparative purposes, all flowers are to the same scale, bar = 1 cm. Flowers were photographed in the wild, in between 7 am to 12 pm, thus the variation is unlikely due to the incomplete opening or senescence. Furthermore, in *ex situ* collection flowers did not change their shape from 7 am to 15 pm. For example, the cup shaped flowers of pink flowered EB139 do not change (Figure 2a), indicating that shown variation is more genetically determined than environmentally controlled. Flowers were arranged according to the curvature of the limb. At the top-left image is a flower shape that we operationally call a cup. The flower series ends with the bottom-right shape called here an umbrella. All flowers were aligned by the proximal end of their corollas. Thus, the next trait the viewer can easily apprehend is variation in flower tube length. Note, that the widest part of the tube is not at a flower throat, as usual in *Ipomoea*, but closer to the middle. In our sampling of 498 flowers, all shapes were not unique in populations. The selected shapes are part of a continuum comprising intermediate shapes, thus we avoid assigning exact shape frequencies.

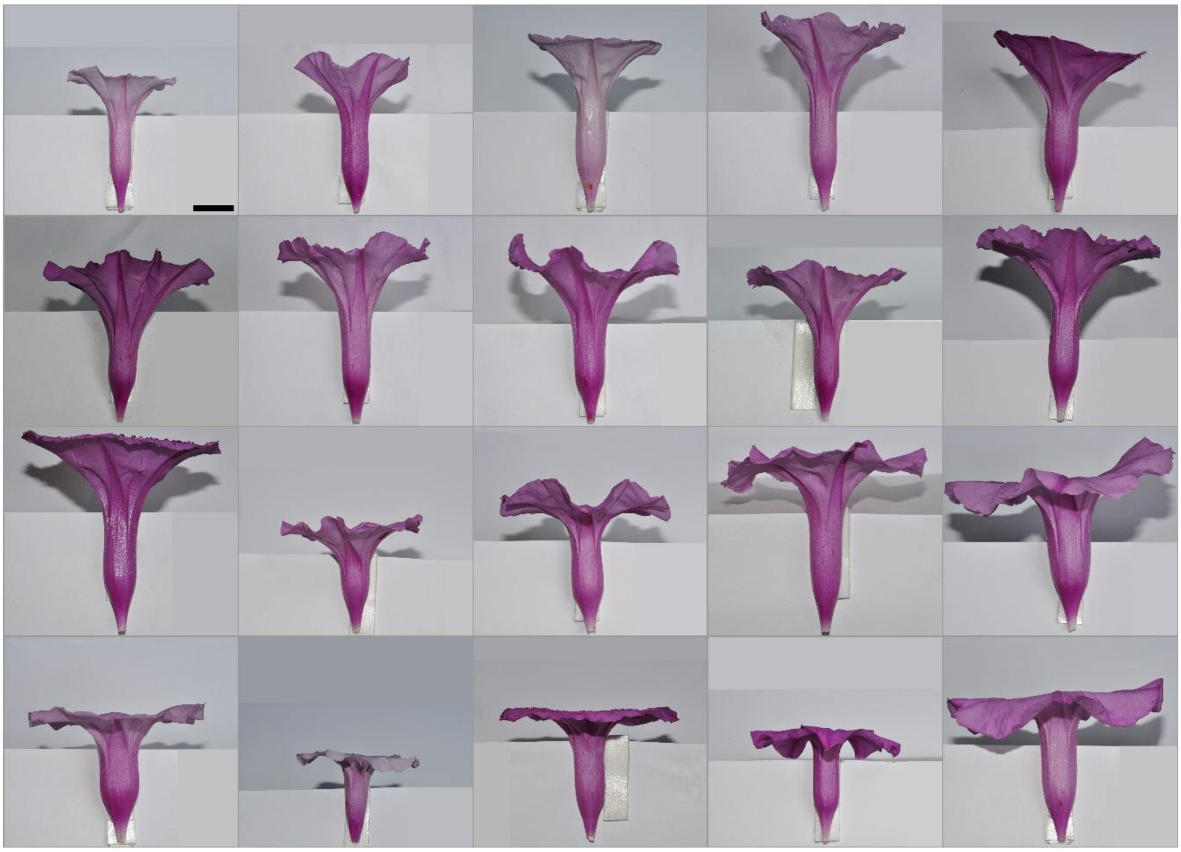

Supplementary Figure S4. Flower shape variation in *I. marabaensis*.

All flowers are to the same scale, bar = 2 cm. Images were selected from a sample of *I. marabaensis* n = 472 from all cangas. Other details as in Supplementary Figure 3.

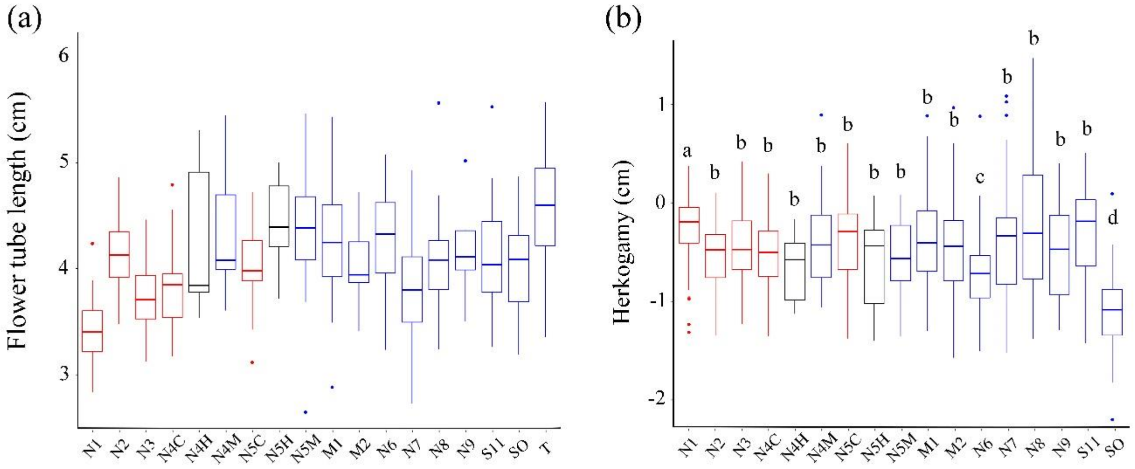

Supplementary Figure S5. Descriptive statistics of the canga-delimited trait diversification illustrated with boxplots.

The band inside the box is a median value; box spans the upper and lower quartiles; box-connected lines are whiskers showing the lowest and highest datum still within 1.5 interquartile range; dots are outliers. Letters above each plot represent statistically significant differences at $p < 0.05$ according to the Wilcoxon post-hoc test. Individual boxplots correspond to canga-delimited samples, the ID's of which are listed along X axis as: cangas N1 to N9; S11 is S11 plateau canga; SO – Sossego granitic inselberg; T – canga Tarzan. Cangas N4 and N5 are sympatric and host putative hybrids "N4H", "N5H"; *I. cavalcantei*, "N4C", "N5C"; and *I. marabaensis*, "N4M", "N5M". Labels "M1" and "M2" identify cangas Morro 1 and Morro 2. Sample species identity is shown by box-plot colouring: red - *I. cavalcantei*; blue - *I. marabaensis*; black – putative hybrids that here are treated as an independent taxon.

(a) Flower tube length. Flower sepals, filaments and styles were removed. The corolla tube length was measured as a distance in cm for the landmark at corolla bottom to the flower throat. Tube length limits access to nectar between visitors that cannot enter the tube, differentiating by the length of tongues.

(b) Herkogamy. Shown are the distances from stigma to the distal tip of the longest stamen in the flower. Values > 0 or < 0, are the ascending and descending herkogamy, respectively.

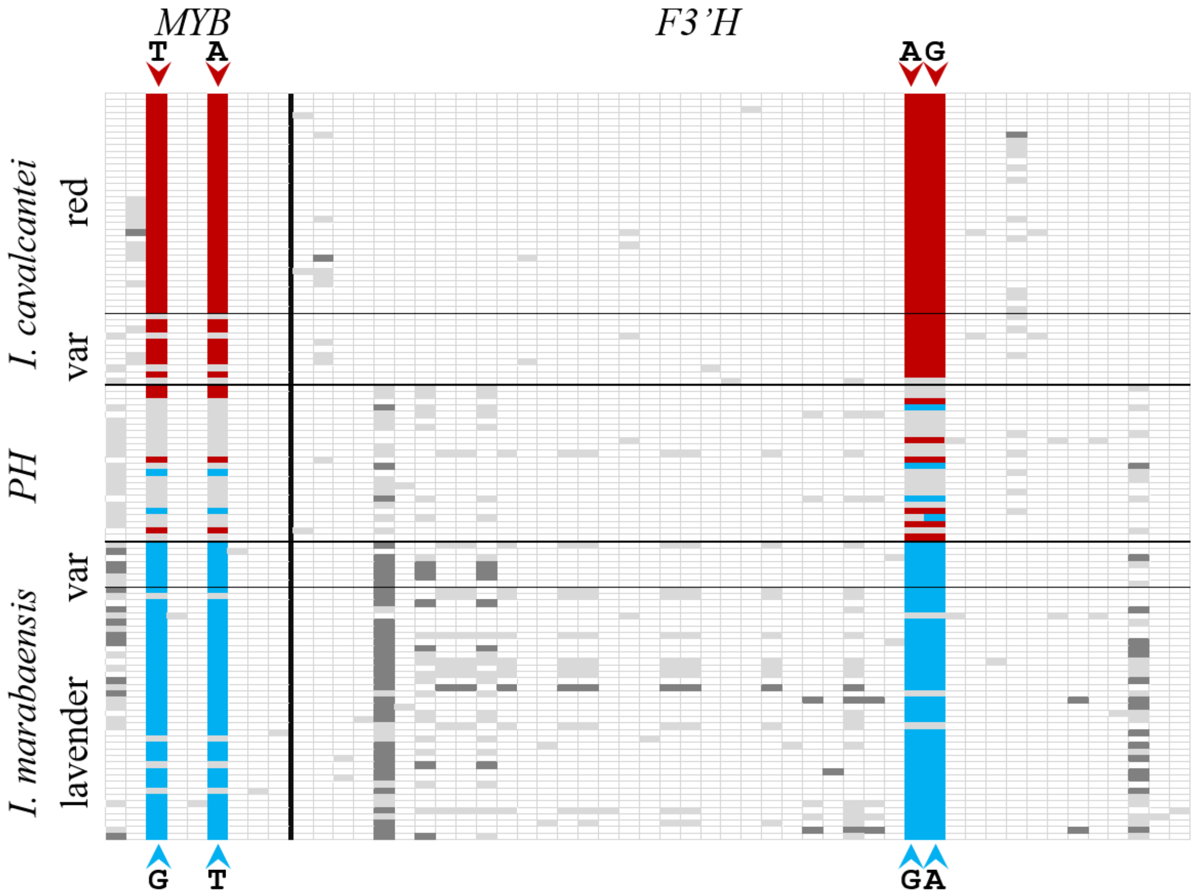

Supplementary Figure S6. Distribution of SNPs in *F3'H* and *MYB* genes.

Partial gene sequences of flavonoid 3' hydroxylase, *F3'H* (465 bp); **My**elo**b**lastosis family transcription factor, *MYB* (598 bp) were analysed for single nucleotide polymorphisms. Columns correspond to homoeologous polymorphic sites, 44 in *F3'H*; 9 in *MYB*. Rows are individuals in DNA sampling. Individuals are grouped by species sampled in all cangas. Subsets of flower colour variants (var) are separated from species standards, red for *I. cavalcantei* and lavender for *I. marabaensis*. Putative hybrids group, *PH*. Cells coloured white represent DNA bases identical in both *I. cavalcantei* and *I. marabaensis*; the alternative bases in homozygous state are dark grey; light grey indicates heterozygosity. The h-SNPs are coloured red or blue, the exact DNA bases are indicated.

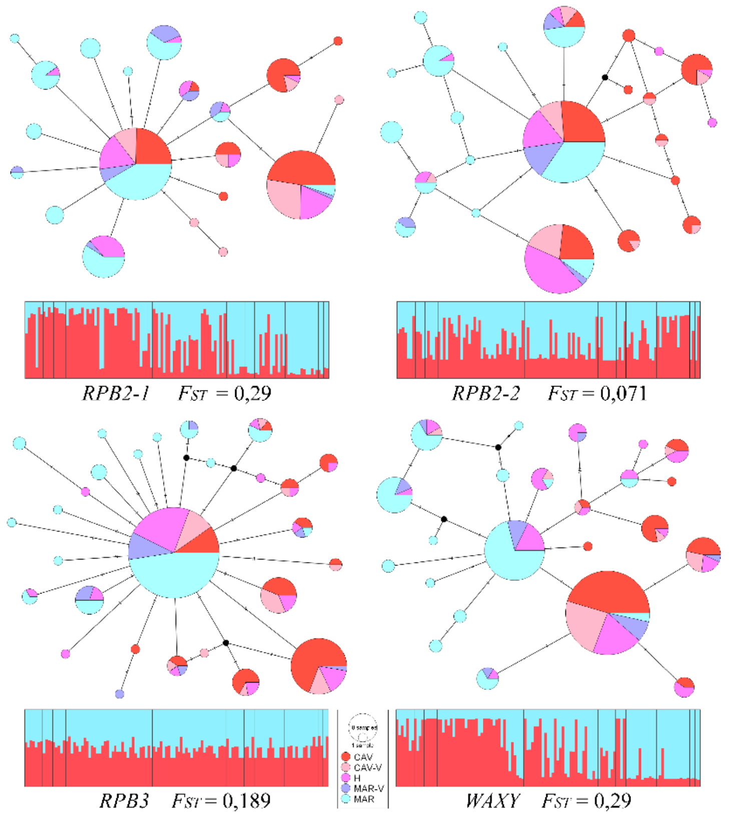

Supplementary Figure S7. Haplotype networks, single locus analysis of admixture (K = 2) and pairwise (*F_{ST}*) interspecies differentiation.

Analysed genes were for subunits of RNA polymerase II, *RPB2-1*, *RPB2-2*, *RPB3*; granule-bound starch synthase, *WAXY*. These results complete those shown the Fig. 3 for all ten genes analysed in this work. INSERT: colouring of haplotype networks: *I. cavalcantei*, CAV; *I. cavalcantei* colour variants, CAV-V; putative hybrids, H; *I. marabaensis* colour variants, MAR-V; *I. marabaensis*, MAR.

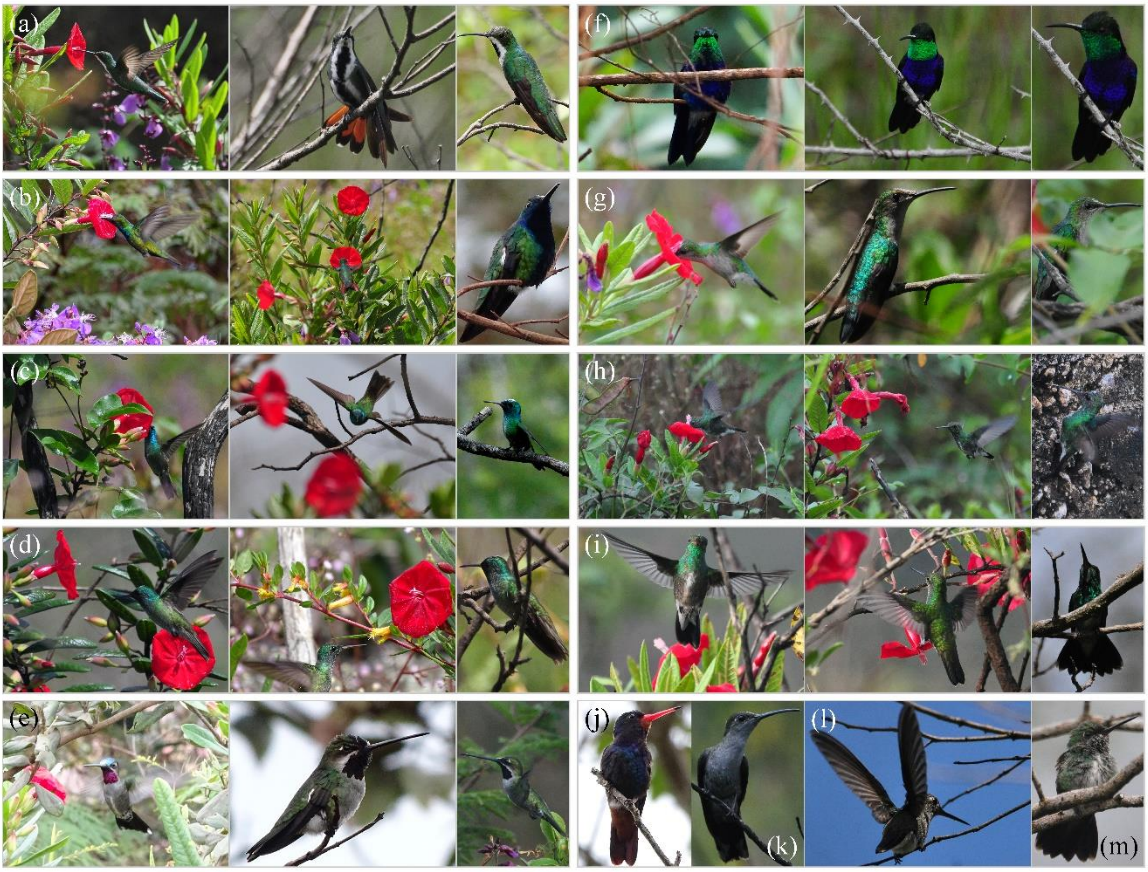

Supplementary Figure S8. Hummingbird species foraging on *I. cavalcantei*.

(a) Female black-throated mango, *Anthracothorax nigricollis*.
(b) Male black-throated mango, *A. nigricollis*.
(c) Male blue-chinned sapphire, *Chlorostilbon notatus*.
(d) Female *C. notatus*.
(e) Male amethyst woodstar, *Calliphlox amethystine*.
(f) Male fork-tailed woodnymph, *Thalurania furcata*.
(g) Female *T. furcata*.
(h,i) *Amazilia fimbriata* and/or *Amazilia versicolor*.
(j) Male rufous-throated sapphire, *Hylocharis sapphirine*.
(k) Gray-breasted sabrewing, *Campylopterus largipennis*.
(l,m) Unidentified species, possibly juveniles. We did not manage to acquire reasonable image of ruby-topaz, *Chrysolampis mosquitus* that was foraging nectar on *I. cavalcantei*.

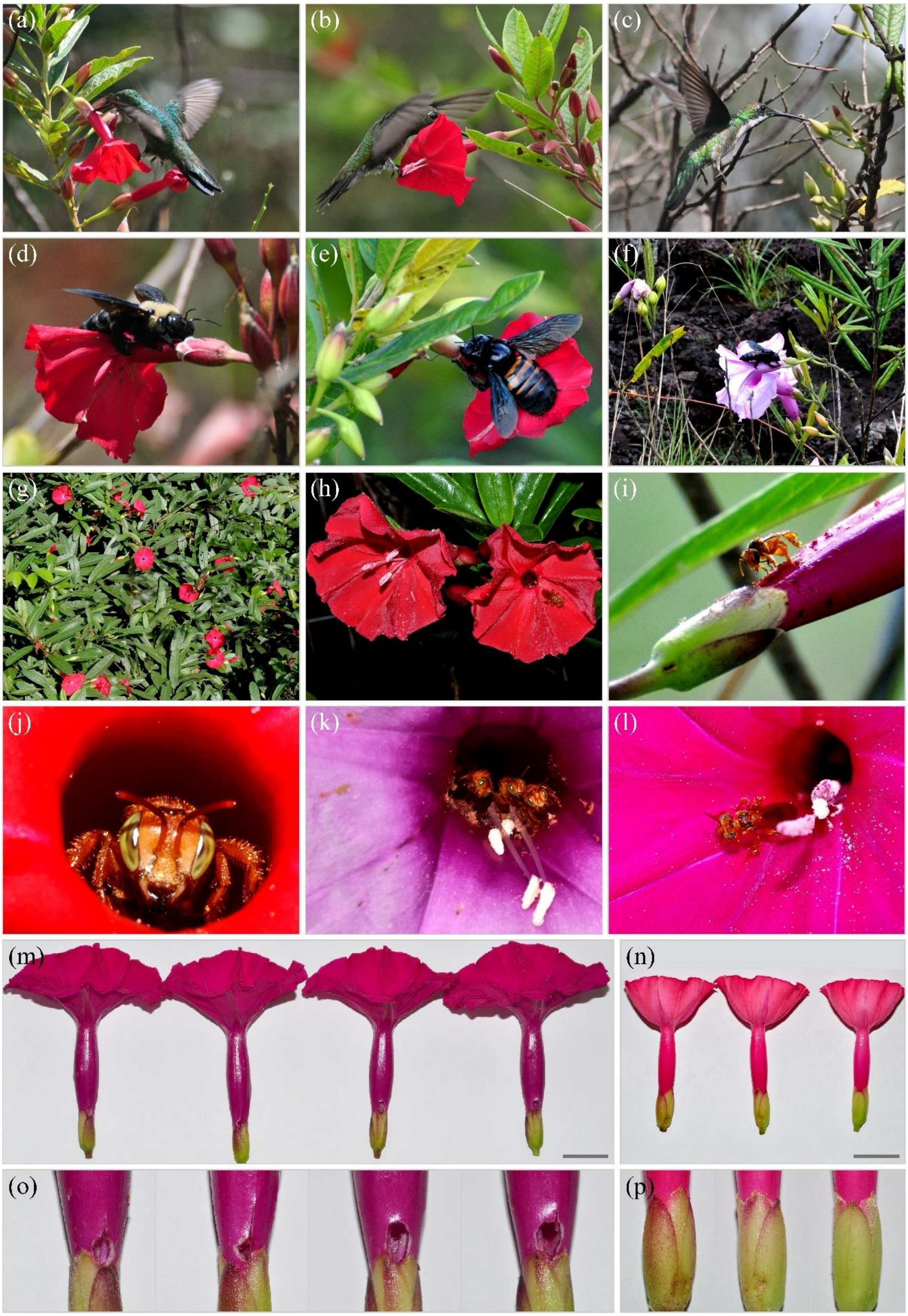

Supplementary Figure S9. Nectar larceny and its effects on plant species reproduction.

(a, b) Nectar robbing by hummingbirds. Hummingbirds use perforations in flower tubes made by carpenter bees to access flower nectar chambers. Hummingbirds stabilize themselves by feet-grabbing onto flower limbs.

(c) Hummingbird forages on a developing fruit capsule of *I. cavalcantei*. Both *I. cavalcantei* and *I. marabaensis* have extrafloral nectaries at leaf petioles, which are commonly visited by ants and wasps. We think that flower sepals, as flower organs closest to leaves in developmental homology, also have ability to produce extrafloral nectar, at least watery secretions were common at sepal bases. Ants, bees and wasps spent time at bases of flower sepals. Nectar produced by sepals in fruit capsule can explain hummingbird visit here.

(d-f) Carpenter bees, *Xylocopa* spp., were very common nectar robbers on *I. cavalcantei* and *I. marabaensis* **(f).**

(g) Black eye (our term) flower phenotype. Throats of six *I. cavalcantei* flowers are visible to the viewer on this image. The lowest to the left flower has whitish spot at the throat, which are exserted style-anthers bundle. The other five flower throats appear as black, i.e. black eye, which is explained by panels **(h)** and **(j)**.

(h) Close up of the black eye phenotype. In a flower to the right, all reproductive organs are absent, a fully developed black eye. In a flower to the left, a style and two filaments are gone. Remaining three filaments are detached at their bases and are being in a process of removal from the flower.

(i) *Trigona pallens* bee and perforation at the flower tube of hybrid EB081 at *ex situ* collection.

(j) *Trigona pallens* at a flower throat. Flower is sterilized by these small bees that remove styles and filament-anthers organs. We explain this behavior as nectar robbing by legitimate access to the flower and followed by breaking into the nectar chamber by demolishing chamber entrance that is formed by the filament bases as described in footnotes to Supplementary Table S14.

(k) *I. marabaensis* flower and *T. pallens* bees. The species flower tubes are broader than in *I. cavalcantei*, and easily accommodate three bees here. Note two filaments-anthers pushed out of the flower.

(l) *T. pallens* bee legitimately access EB081 hybrid flower. Only two anthers and style are remaining at that moment.

(m, o) Flowers of EB081 produced on a given day. All four flowers tube bases have perforations made by *T. pallens* bees, i.e. four flower a day - all damaged.

(n, p) Nectar wells. Three flowers of *I. cavalcantei* pink-flowered plant EB139. In **(p)**, nectar wells (our term) are near the flower bases of two flowers to the left. This is a third route, which we think is the smartest, used by the *T. pallens* bees to access the nectar. Bees carve out flower tissues through the sepals. Once a bee reaches the nectar chamber, it stops, at least we have not seen any damage to the carpels. Many insects, including honeybees, visit nectar wells made by *T. pallens*. The nectar wells are very common and corresponding circular scars are easily recognizable on fruit capsules.

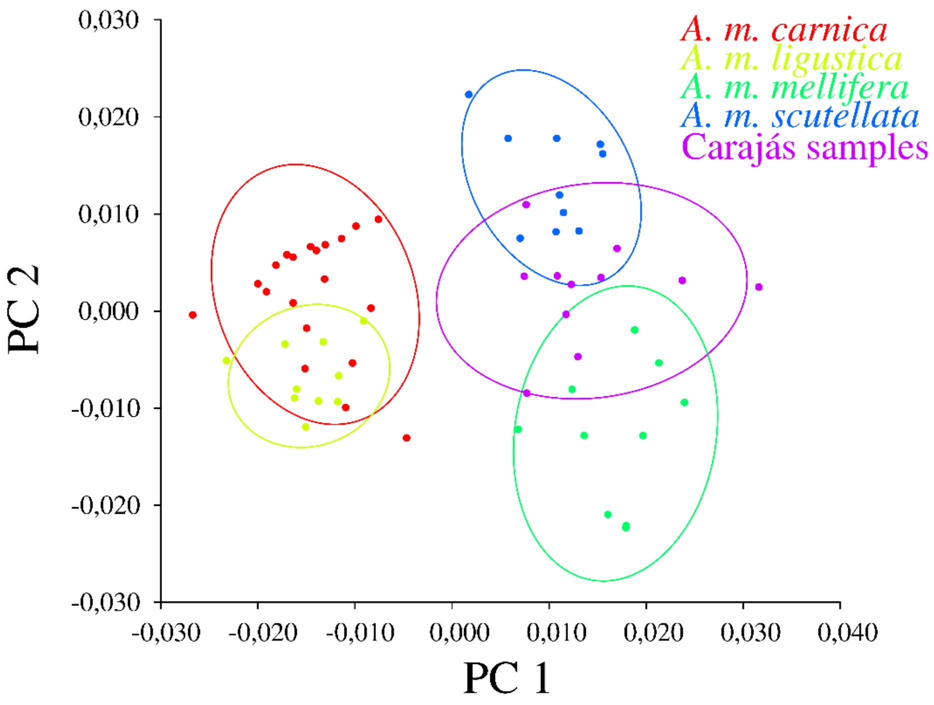

Supplementary Figure S10. Honeybee race identification.

To determine which subspecies of *Apis mellifera* was visiting the *Ipomoea* flowers, we used 19 anatomical landmarks at the junctions of the veins of the right anterior wings[75]. The honey bees collected in Carajás were compared with samples of the following subspecies: *A. m. carnica, A. m. ligustica, A. m. mellifera* and *A. m. scutellata*, obtained from the Ruttner Collection - Morphometric Bee Data Bank in Oberursel, Germany. After Procrustes fit, a PCA was performed in software MorphoJ[112]. According to the result of the PCA analysis in comparison to previous data (Francoy, et al. 2008; 2009), the *Apis mellifera* collected in Carajás present intermediate wing shape between *Apis mellifera mellifera* and *Apis mellifera scutellata*, indicating that it is the so-called Africanized honey bees that visited *I. cavalcantei* flowers as recorded in our field studies. The bees were photographed in dorsal view with a photographic camera coupled to a stereoscopic microscope. The intertegular distance of floral visitors was measured by establishing a straight line connecting the two tegulas (wing insertion), using ImageJ software version 1.52a (https://imagej.nih.gov/ij/docs/guide/146.html)[115,116].

Francoy, T. M., Wittmann, D., Drauschke, M., Müller, S., Steinhage, V., Bezerra-Laure, M. A. & Gonçalves, L. S. Identification of Africanized honey bees through wing morphometrics: two fast and efficient procedures. *Apidologie* **39**, 488-494 (2008).

Francoy, T. M., Wittmann, D., Steinhage, V., Drauschke, M., Müller, S., Cunha, D. R. & Arias, M. C. (2009). Morphometric and genetic changes in a population of Apis mellifera after 34 years of Africanization. *Genet. Mol. Res.* **8**, 709-717 (2009).

Supplementary Table S1. Study locations.

Canga	Sites	LAT (DMS)	LON (DMS)	Site Access[a]
N1	SITE1	-6° 0' 55.8"	-50° 17' 48.1"	2 permits; 4WD car, easy
N1	SITE2	-6° 1' 46.2"	-50° 17' 6.2"	ibid
N1	SITE3	-6° 1' 51.9"	-50° 16' 60"	ibid
N1	SITE4	-6° 1' 40.8"	-50° 16' 37.7"	ibid
N1	SITE5	-6° 2' 0.1"	-50° 17' 3.2"	ibid
N1	SITE6	-6° 2' 40.2"	-50° 16' 3.8"	ibid
N1	SITE7	-6° 0' 56.6"	-50° 17' 43.9"	ibid
N2	SITE1	-6° 3' 28.6"	-50° 15' 2.2"	2 permits; 4WD car, easy
N2	SITE2	-6° 3' 28.3"	-50° 15' 3.3"	ibid
N2	SITE3	-6° 3' 25"	-50° 15' 0.4"	ibid
N2	SITE4	-6° 3' 16.9"	-50° 15' 3.6"	ibid
N2	SITE5	-6° 3' 28.6"	-50° 14' 42"	ibid
N2	SITE6	-6° 3' 9.7"	-50° 15' 4.3"	ibid
N2	SITE7	-6° 3' 32.8"	-50° 14' 44.3"	ibid
N3	SITE1	-6° 2' 43.4"	-50° 13' 8.5"	2 permits; 4WD car, easy
N3	SITE2	-6° 2' 47.2"	-50° 13' 9.7"	ibid
N3	SITE3	-6° 2' 21.7"	-50° 12' 49.7"	ibid
N3	SITE4	-6° 2' 30.7"	-50° 12' 36"	ibid
N3	SITE5	-6° 2' 29.4"	-50° 12' 39.3"	ibid
N3	SITE6	-6° 2' 27.1"	-50° 12' 41.2"	ibid
N3	SITE7	-6° 2' 21.1"	-50° 12' 50.8"	ibid
N4	SITE1	-6° 5' 41.1"	-50° 11' 41"	3 permits; 4WD car, moderate
N4	SITE2	-6° 6' 30.9"	-50° 11' 14.6"	ibid
N4	SITE3	-6° 6' 45.1"	-50° 11' 2.9"	ibid
N4	SITE4	-6° 6' 45.6"	-50° 11' 1.6"	ibid
N4	SITE5	-6° 4' 21.8"	-50° 11' 35.5"	ibid
N4	SITE6	-6° 6' 34.8"	-50° 11' 12.3"	ibid
N4	SITE7	-6° 6' 42.7"	-50° 11' 2.8"	ibid
N4	SITE8	-6° 6' 34.9"	-50° 11' 1.6"	ibid
N5	SITE1	-6° 5' 21.5"	-50° 8' 28.1"	3 permits; 4WD car, difficult
N5	SITE2	-6° 5' 12.2"	-50° 7' 28"	ibid
N5	SITE3	-6° 5' 17.5"	-50° 8' 18.8"	ibid
N5	SITE4	-6° 5' 10.8"	-50° 7' 29.6"	ibid
N5	SITE5	-6° 5' 15.6"	-50° 7' 30.8"	ibid
N5	SITE6	-6° 5' 19.8"	-50° 7' 27.3"	ibid
N6	SITE1	-6° 7' 47.6"	-50° 10' 36.1"	3 permits; 4WD car, difficult
N6	SITE2	-6° 7' 49.4"	-50° 10' 36.8"	ibid
N6	SITE3	-6° 7' 50"	-50° 10' 33.7"	ibid
N6	SITE4	-6° 7' 47.8"	-50° 10' 33.8"	ibid
N6	SITE5	-6° 7' 44.5"	-50° 10' 31.8"	ibid
N6	SITE6	-6° 7' 54.2"	-50° 10' 26.5"	ibid
N6	SITE7	-6° 7' 48.7"	-50° 10' 26.2"	ibid

N7	SITE1	-6° 9' 8"	-50° 10' 32.7"	3 permits; 4WD car, difficult
N7	SITE2	-6° 9' 10.3"	-50° 10' 29.2"	ibid
N7	SITE3	-6° 9' 11.7"	-50° 10' 28.2"	ibid
N7	SITE4	-6° 9' 13.2"	-50° 10' 25.6"	ibid
N7	SITE5	-6° 9' 27.1"	-50° 10' 16.6"	ibid
N7	SITE6	-6° 9' 22.8"	-50° 10' 16.4"	ibid
N8	SITE1	-6° 10' 26.6"	-50° 8' 59.7"	3 permits; 4WD car, difficult
N8	SITE2	-6° 10' 21.9"	-50° 9' 7.3"	ibid
N8	SITE3	-6° 10' 15"	-50° 9' 15.9"	ibid
N8	SITE4	-6° 10' 8.6"	-50° 9' 24.1"	ibid
N8	SITE5	-6° 10' 50.7"	-50° 8' 30.1"	ibid
N8	SITE6	-6° 9' 54.3"	-50° 9' 46.2"	ibid
M1	SITE1	-6° 6' 28"	-50° 8' 24.7"	2 permits; 4WD car, easy
M1	SITE2	-6° 6' 26.1"	-50° 8' 18.7"	ibid
M1	SITE3	-6° 6' 44.9"	-50° 8' 17"	ibid
M2	SITE1	-6° 7' 30.4"	-50° 8' 1.3"	2 permits; 4WD car, easy
M2	SITE2	-6° 7' 6.9"	-50° 7' 58.8"	ibid
N9	SITE1	-6° 11' 5.2"	-50° 8' 3.3"	3 permits; 4WD car, difficult
S11	SITE1	-6° 23' 42.8"	-50° 22' 23.3"	2 permits; 4WD car, time costly
S11	SITE2	-6° 23' 48.7"	-50° 22' 22.4"	ibid
S11	SITE3	-6° 22' 58.4"	-50° 23' 6.4"	ibid
S11	SITE4	-6° 22' 46.5"	-50° 22' 58.1"	ibid
S11	SITE5	-6° 20' 58.9"	-50° 26' 56.5"	ibid
S11	SITE6	-6° 20' 57.6"	-50° 26' 55.5"	ibid
S11	SITE7	-6° 20' 59"	-50° 26' 51.9"	ibid
SO	SITE1	-6° 27' 34.6"	-50° 5' 8.8"	2 permits; 4WD car, time costly
SO	SITE2	-6° 27' 47"	-50° 5' 19.2"	ibid
SO	SITE3	-6° 27' 37.2"	-50° 5' 7.1"	ibid
T	SITE1	-6° 19' 57.0"	-50° 9' 54.0"	2 permits; helicopter, money costly
T	SITE2	-6° 20' 3.8"	-50° 9' 57.2"	ibid
T	SITE3	-6° 20' 12.0"	-50° 9' 53.0"	ibid

[a] The field work and sampling of native biodiversity requires an authorization by the SISBIO (http://www.icmbio.gov.br/sisbio/); Chico Mendes Institute of Biodiversity Conservation (ICMBio); Brazilian Ministry of Environment (MMA). The second permit, renewable every five days, to access Carajás National Forest must be acquired from VALE S.A. multinational mining company. The third permit is dictated by the mining operations: (i) if site is accessible only through the mine then a guide scout car, pending availability, is obligatory (e.g. canga N5 sites); (ii) if canga (e.g. N4) or the access road to canga (N6, N7, N8) is at the periphery of a mine additional clearance is required. Four-wheel drive (4WD) car is necessary to access cangas, using dirt track roads laid down by geological surveys. Falling trees and road erosion due to rain increase the difficulty (difficult) of access to N4, N6, N7, N8, N9. Access to S11 Plateau through the forest is time consuming. Access to Tarzan is mainly by money costly helicopter, pending availability and weather conditions.

Supplementary Table S2. Sample sizes (n) in canga-delimited populations.

Sample ID[a]	Flower geometric morphometrics analysis	Descriptive statistics		
		Flower	Leaf	Reproductive organs
N1	133	127	60	92
N2	130	130	41	64
N3	124	123	28	90
N4C	95	93	75	82
N4H	7	7	4	5
N4M	11	10	11	25
N5C	25	25	39	30
N5H	27	28	28	8
N5M	27	30	32	12
M1	67	69	41	33
M2	20	21	36	45
N6	120	53	49	44
N7	101	60	28	45
N8	61	66	35	56
N9	6	8	20	10
S11	133	68	53	39
SO	58	34	37	45
T	43	53	69	NA

[a] Cangas codes as accepted by geological surveys; abbreviated are: Morro 1, M1; Morro 2, M2; S11 Plateau, S11; Sossego, SO; Tarzan, T. In sympatric cangas N4 and N5, samples representing *I. cavalcantei*, C; *I. marabaensis*, M; putative hybrids, H. NA – no sampling.

Supplementary Table S3. Trait measurements for the species and putative hybrids.

	I. cavalcantei[e]		*I. marabaensis*[e]		Putative hybrids[e]	
	Range	Mean ± SD	Range	Mean ± SD	Range	Mean ± SD
Diameter of flower[a,b]	2.92-7.98	4.50±0.58	4.45-11.67	8.12± 1.05	4.59-8.28	6.54±0.94
Throat width[a,b]	0.33-0.92	0.47±0.05	0.85-2.24	1.4 ±0.18	0.50-1.17	0.81±0.16
Tube-limb angle [c,d]	66.08-134.82	106.4±11.2	103-157.3	135.5±9.03	96.43-137.43	111.11±14.9
Tube length[a,c]	2.83-4.86	3.80±0.42	2.48-5.57	4.16±0.53	3.53-6.05	4.48±0.61
Included/exserted style[a]	(-0.08)-1.94	0.61±0.454	(-3.6)-(-0.15)	(-1.5)±0.64	(-2.1)-0.7	(-0.41)±0.73
Herkogamy[a]	(-1.38)-0.603	(-0.43)±0.34	(-2.2)-1.47	(-0.5)±0.58	(-1.4)-0.07	(-0.67)±0.47

[a] Values in centimetres (cm).
[b] Measurements from the frontal view imagery.
[c] Measurements from the lateral view imagery.
[d] Values are in degrees; an angle between tube axis and limb plane.
[e] Sample size for flower corolla characters: *I. cavalcantei* n = 498; *I. marabaensis* n = 472; putative hybrids n = 35. Sample size for reproductive organs: *I. cavalcantei* n = 358; *I. marabaensis* n = 354; putative hybrids n = 12.

Supplementary Table S4. Summary of shoot isolation experiments in a wild.

I. cavalcantei				*I. marabaensis*			
Canga N1		Canga N4[a]		Canga N6		Canga N8	
Plant	Abscised flowers/ Ripening capsules	Plant	Abscised flowers/ Ripening capsules	Plant	Abscised flowers/ Ripening capsules	Plant	Abscised flowers/ Ripening capsules
#1	16/0	#1	15/0	#1	26/0	#1	12/0
#2	21/0	#2	17/0	#2	10/0	#2	19/0
#3	46/0	#3	11/0	#3	13/0	#3	11/0
#4	56/0	#4	9/0	#4	21/0	#4	17/0
#5	18/0	#5	2/0	#5	18/0	#5	14/0
#6[b]	27/0	#6	10/0	#6	16/0	#6	16/0
#7[b]	32/0	#7	17/0	#7	14/0	#7	13/0
#8	16/0	#8	13/0	#8	19/0	#8	14/0
#9	43/0	#9	8/0	#9	11/0	#9	25/0
#10	14/0	#10	4/0	#10	23/0	#10	15/0
#11[b]	19/0	#11	13/0	#11	14/0	#11	28/0
#12[b]	44/0	#12	NA	#12	24/0	#12	24/0
#13	26/0	#13	NA	#13	17/0	#13	26/0
#14	37/0	#14	NA	#14	21/0	#14	19/0
#15	53/0	#15	NA	#15	29/0	#15	28/0
#16	16/0						
Total	484/0		119/0		276/0		281/0

[a] Bagged shoots from four plants were found broken off the plant. Those were not analysed (NA).
[b] Isolated shoots of these plants continued to flower at time of collection.

Supplementary Table S5. Controlled self-pollinations of individuals in *ex situ* collection.

Line	Species; flower colour [b]	Self-pollinated flowers	Fruit capsules
EBD1	C; red	2	0
EB148	C; red	2	0
EBD2	C; red	2	0
EB011	C; white	3	0
EB139	C; pink	4	0
EB145	C; pink	2	0
EB079	C; pink	4	0
EB048	C; pink	4	0
EB147	C; purplish	2	0
EB123	C; purplish	2	0
EBPZB1	M; lavender	3	0
EB129	M; pale lavender	2	0
EB024	M; white	9	0
EB081	H; magenta	4	0
EB022	H; magenta	1	0
EB015	H; magenta	1	0
EB088	H; magenta	1	0

[a] Plants grown *ex situ* in Vale Parco Zoobotanico were used in controlled pollination experiments. All tested flowers were isolated using cheesecloth bag technique as described in Methods. When young capsules abscised, i.e. fell off the mother plant, within four to seven days after self-pollination pollination was scored as failed. To reduce the effect of physical damage to styles due to emasculation, the results shown are with intact flowers, i.e. pollen is from the same flower. On a putative hybrid EB081 and *I. cavalcantei* EBD1 we also used emasculated flowers pollinated with pollen of the same plants, i.e. to test geitonogamy. Those crosses were not successful as well.

[b] *I. cavalcantei* (C), *I. marabaensis* (M) and putative hybrids (H) were tested. Variation in flower limb colour is indicated.

Supplementary Table S6. Progeny analysis.

		ANS			*MYB*			*UFGT*			*WD40*			
Plant	ID	CHR1	CHR2	P[b]	CHR1	CHR2	P[b]	CHR1	CHR2	P[b]	CHR1	CHR2	P[b]	Cross[c]
**Mother**	**EB139 C pink**	H05	H05		H02	H03		H04	H06		H04	H16		
Progeny	EB139 PR1	H05	H01	O	H02	H03	S/O	H04	H06	S/O	H04	H05	O	OUTCROSS
Progeny	EB139 PR2	H05	H25	O	H02	H02	S/O	H04	H06	S/O	H16	H05	O	OUTCROSS
Progeny	EB139 PR3	H05	H15	O	H02	H03	S/O	H06	H06	S/O	H04	H15	O	OUTCROSS
Progeny	EB139 PR4	H05	H19	O	H02	H02	S/O	H04	H04	S/O	H16	H05	O	OUTCROSS
Progeny	EB139 PR5	H05	H02	O	H02	H03	S/O	H04	H04	S/O	H04	H04	S/O	OUTCROSS
Progeny	EB139 PR6	H05	H01	O	H02	H03	S/O	H04	H06	S/O	H04	H14	O	OUTCROSS
**Mother**	**EB017 C pink**	H01	H21		H01	H02		H04	H04		H05	H05		
Progeny	EB017 PR1	H01	H21	S/O	H02	H02	S/O	H04	H06	O	H05	H06	O	OUTCROSS
Progeny	EB017 PR2	H01	H16	O	H02	H03	O	H04	H01	O	H05	H06	O	OUTCROSS
**Mother**	**EB019 C pink**	H01	H21		H02	H04	S/O	H06	H06		H02	H02		
Progeny	EB019 PR1	H21	H02	O	H02	H04	S/O	H06	H15	O	H02	H06	O	OUTCROSS
Progeny	EB019 PR2	H01	H21	S/O	H04	H04	S/O	H06	H15	O	H02	H06	O	OUTCROSS
Progeny	EB019 PR3	H01	H02	O	H02	H04	S/O	H06	H06	S/O	H02	H06	O	OUTCROSS
Progeny	EB019 PR4	H01	H02	O	H02	H02	S/O	H06	H01	O	H02	H10	O	OUTCROSS
**Mother**	**EB079 C pink**	H02	H13		H02	H03		H01	H04		H04	H06		
Progeny	EB079 PR1	H13	H13	S/O	H02	H04	O	H04	H03	O	H04	H19	O	OUTCROSS
Progeny	EB079 PR2	H02	H15	O	H02	H04	O	H01	H03	O	H06	H07	O	OUTCROSS
Progeny	EB079 PR3	H02	H15	O	H02	H04	O	H01	H02	O	H04	H19	O	OUTCROSS
Progeny	EB079 PR4	H02	H21	O	H02	H03	S/O	H01	H04	S/O	H06	H05	O	OUTCROSS
Progeny	EB079 PR5	H02	H13	O	H02	H03	S/O	H01	H04	S/O	H04	H05	O	OUTCROSS
**Mother**	**EB126 C pur**	H02	H21		H02	H04		H01	H01		H02	H06		
Progeny	EB126 PR1	H02	H21	S/O	H02	H03	O	H01	H04	O	H02	H04	O	OUTCROSS
Progeny	EB126 PR2	H21	H01	O	H02	H03	O	H01	H04	O	H02	H02	S/O	OUTCROSS
**Mother**	**EB011 C w**	H02	H21		H02	H02		H04	H04		H06	H06		
Progeny	EB011 PR1	H02	H41	O	H02	H02	S/O	H04	H04	S/O	H06	H05	O	OUTCROSS
**Mother**	**EB138 H**	H01	H01		H01	H04		H04	H06		H04	H11		
Progeny	EB138 PR1	H01	H13	O	H04	H04	S/O	H06	H10	O	H04	H07	O	OUTCROSS
Progeny	EB138 PR2	H01	H11	O	H01	H04	S/O	H04	H06	S/O	H11	H07	O	OUTCROSS
**Mother**	**EB015 H**	H01	H11		H02	H04		H01	H06		H02	H04		
Progeny	EB015 PR1	H01	H02	O	H02	H04	S/O	H01	H01	S/O	H02	H06	O	OUTCROSS
Progeny	EB015 PR2	H01	H02	O	H02	H02	S/O	NA	NA	NA	H02	H02	S/O	OUTCROSS
Progeny	EB015 PR3	H01	H01	S/O	H02	H02	S/O	H01	H06	S/O	H04	H06	O	OUTCROSS
Progeny	EB015 PR4	H11	H02	O	H02	H04	S/O	H01	H01	S/O	H02	H04	S/O	OUTCROSS
Progeny	EB015 PR5	H01	H11	S/O	H02	H04	S/O	H01	H06	S/O	H02	H06	O	OUTCROSS
**Mother**	**EB047 H**	H11	H21		H02	H04		H04	H04		H04	H03		
Progeny	EB047 PR1	H11	H13	O	H02	H04	S/O	H04	H03	O	H03	H02	O	OUTCROSS
Progeny	EB047 PR2	H21	H13	O	H02	H04	S/O	H04	H03	O	H04	H02	O	OUTCROSS
**Mother**	**EB081 H**	H15	H17		H02	H02		H03	H06		H07	H07		
Progeny	EB081 PR1	H17	H33	O	H02	H02	S/O	H03	H01	O	H07	H04	O	OUTCROSS
Progeny	EB081 PR2	H15	H03	O	H02	H02	S/O	H03	H01	O	H07	H04	O	OUTCROSS
Progeny	EB081 PR3	H17	H17	S/O	H02	H01	O	H06	H27	O	H07	H07	S/O	OUTCROSS
Progeny	EB081 PR4	H15	H15	S/O	H02	H02	S/O	H06	H01	O	H07	H16	O	OUTCROSS
Progeny	EB081 PR5	H17	H02	O	H02	H02	S/O	H06	H06	S/O	H07	H07	S/O	OUTCROSS
Progeny	EB081 PR6	H15	H41	O	H02	H03	O	H06	H06	S/O	H07	H04	S/O	OUTCROSS
Progeny	EB081 PR7	H15	H02	O	H02	H03	O	H06	H03	S/O	H07	H04	S/O	OUTCROSS
Progeny	EB081 PR8	H15	H41	O	H02	H02	S/O	H06	H06	S/O	H07	H02	O	OUTCROSS
Progeny	EB081 PR9	H15	H15	S/O	H02	H03	O	H03	H01	O	H07	H04	S/O	OUTCROSS
Progeny	EB081 PR10	H15	H21	O	H02	H03	O	H03	H01	O	H07	H04	S/O	OUTCROSS

[a] Seeds were collected from indicated mother plants that grew in sympatric canga N4, except EB011 white flowered individual from N3. *I. cavalcantei* flower colour variants were pink-flower (C pink), purplish (C pur) and white (C w). Putative hybrids (H). Seeds were germinated and resulting progeny was raised in growth chambers. DNA was extracted from preserved leaf tissues of mothers and fresh leaves from progeny (PR) plants. Genotypes were identified by sequencing amplicons of four genes (*ANS, MYB, UFGT, WD40*). Gene alleles, i.e., haplotypes (H), on chromosomes (CHR1, CHR2) are listed. NA – not analysed.

[b] Those columns (P) explain the genotype of progeny at a given locus as due to outcross (O), or either due to self-pollination or outcross (S/O). The S/O events are ambiguous in regard of the pollen donors.

[c] This column (Cross) summarizes the genotyping at all four loci. The progeny plant was considered as the result of pollination from a different individual than a mother (OUTCROSS) only when the plant was scored (O) at least at one locus, i.e. one O overrides all the ambiguous S/O.

Supplementary Table S7. Intraspecific crosses.

Summary of intraspecific crosses				
Cross[a]	Parental combinations total/compatible[b]	Flower/fruits[c]	Seeds[d]	Seed set[e]
C×C	19/10	34/19	62	82 %
M×M	7/4	12/5	17	85%
**List of individual intraspecific crosses[f]**				
**Style**	**Pollen**	**Flower[c]**	**Cross[a]**	**Seeds[d]**
EB048 (C/P/N4)	CAV_PZB4 (C/R/N4)	ABORTED	C×C	0
EB048 (C/P/N4)	CAV_PZB5 (C/R/N4)	CAPSULE	C×C	4
EB123 (C/U/N4)	CAV_PZB1 (C/R/N4)	ABORTED	C×C	0
EB123 (C/U/N4)	CAV_PZB2 (C/R/N4)	CAPSULE	C×C	4
EB123 (C/U/N4)	CAV_PZB3 (C/R/N4)	ABORTED	C×C	0
EB139 (C/P/N4)	CAV_PZB1 (C/R/N4)	ABORTED	C×C	0
EB139 (C/P/N4)	CAV_PZB1 (C/R/N4)	ABORTED	C×C	0
EB139 (C/P/N4)	CAV_PZB1 (C/R/N4)	ABORTED	C×C	0
EB139 (C/P/N4)	CAV_PZB2 (C/R/N4)	CAPSULE	C×C	3
EB139 (C/P/N4)	CAV_PZB2 (C/R/N4)	CAPSULE	C×C	2
EB139 (C/P/N4)	CAV_PZB2 (C/R/N4)	CAPSULE	C×C	4
EB139 (C/P/N4)	CAV_PZB3 (C/R/N4)	ABORTED	C×C	0
EB139 (C/P/N4)	CAV_PZB3 (C/R/N4)	ABORTED	C×C	0
EB139 (C/P/N4)	CAV_PZB3 (C/R/N4)	ABORTED	C×C	0
EB139 (C/P/N4)	CAV_PZB3 (C/R/N4)	ABORTED	C×C	0
EB139 (C/P/N4)	CAV_PZB4 (C/R/N4)	CAPSULE	C×C	4
EB139 (C/P/N4)	CAV_PZB4 (C/R/N4)	CAPSULE	C×C	4
EB139 (C/P/N4)	CAV_PZB5 (C/R/N4)	CAPSULE	C×C	3
EB139 (C/P/N4)	CAV_PZB5 (C/R/N4)	CAPSULE	C×C	4
EB139 (C/P/N4)	EB048 (C/P/N4)	CAPSULE	C×C	2
EB139 (C/P/N4)	EB048 (C/P/N4)	CAPSULE	C×C	2
EB139 (C/P/N4)	EB079 (C/P/N4)	CAPSULE	C×C	3
EB139 (C/P/N4)	EB079 (C/P/N4)	CAPSULE	C×C	3
EB139 (C/P/N4)	EB145 (C/P/N4)	ABORTED	C×C	0
EB139 (C/P/N4)	EB145 (C/P/N4)	CAPSULE	C×C	4
EB139 (C/P/N4)	EBD1 (C/R/N4)	CAPSULE	C×C	4
EB079 (C/P/N4)	EB148 (C/R/N4)	ABORTED	C×C	0
EB079 (C/P/N4)	EBD1 (C/R/N4)	ABORTED	C×C	0
EB079 (C/P/N4)	EBD1 (C/R/N4)	ABORTED	C×C	0
EB079 (C/P/N4)	EBD1 (C/R/N4)	ABORTED	C×C	0
EBD1 (C/R/N4)	EB123 (C/U/N4)	CAPSULE	C×C	3
EBD1 (C/R/N4)	EB139 (C/P/N4)	CAPSULE	C×C	3
EBD1 (C/R/N4)	EB139 (C/P/N4)	CAPSULE	C×C	3
EBD2 (C/R/N4)	EB139 (C/P/N4)	CAPSULE	C×C	3
EB024 (M/W/N6)	EB156 (M/L/N8)	ABORTED	M×M	0
EB024 (M/W/N6)	EB156 (M/L/N8)	ABORTED	M×M	0
EB024 (M/W/N6)	EB156 (M/L/N8)	ABORTED	M×M	0
EB024 (M/W/N6)	EBPZB1 (M/L/S11)	ABORTED	M×M	0

EB024 (M/W/N6)	EBPZB1 (M/L/S11)	CAPSULE	M×M	2
EB024 (M/W/N6)	MAR_SO1 (M/L/SO)	CAPSULE	M×M	4
EB024 (M/W/N6)	MAR_SO1 (M/L/SO)	CAPSULE	M×M	4
EB024 (M/W/N6)	MAR_SO2 (M/L/SO)	ABORTED	M×M	0
EB024 (M/W/N6)	MAR_SO2 (M/L/SO)	ABORTED	M×M	0
EB129 (M/L/N6)	EB156 (M/L/N8)	ABORTED	M×M	3
EB129 (M/L/N6)	EBPZB1 (M/L/S11)	CAPSULE	M×M	4
EB129 (M/L/N6)	MAR_SO1 (M/L/SO)	ABORTED	M×M	0

[a] *I. cavalcantei* × *I. cavalcantei* (C×C); *I. marabaensis* × *I. marabaensis* (M×M).
[b] Total number of parental combinations tested versus the combinations that produced offspring, i.e., compatible parental combinations.
[c] Number of pollinated flowers versus developed fruit capsules. For some parental combinations several pollinations had been performed, thus the numbers of pollinated flowers are larger than the number of tested parental combination. In the list of tested parental combination, the column (Flower) summarizes the fate of pollinated flower, which in some parental combinations was aborted within 3-7 days flowers (ABORTED), or developed into a fruit capsule (CAPSULE).
[d] Number of seeds produced from harvested fruit capsules.
[e] Seed set efficiency was calculated as percentage of harvested seeds from the maximal expected seed set following a rule, one fruit capsule generally produces four seeds.
[f] Direction of the cross is in columns (Style) and (Pollen), mother and father, respectively. Individual plant identification numbers followed by three explanations in brackets, i.e., (i) species *I. cavalcantei* (C), *I. marabaensis* (M); (ii) flower colour, red (R), pink (P), purplish (U), lavender (L), white limb (W); (iii) canga origin N4 (N4), N6 (N6), N8 (N8), S11 Plateau (S11), Sossego (SO).

Supplementary Table S8. Genetic diversity indices and pair-wise population differentiation.

Gene	Species[a]	bp[b]	PS[c]	HAP[d]	He obs[e]	He exp[e]	π^e	Tajima's D	Fu's *Fs*	Fst[f]	
RPB2-2	M	678	15	14	0.56	0.82	0.0033	-0.753; P = 0.261	-3.30; P = 0.11	0.071	
RPB2-2	C	678	12	12	0.81	0.80	0.0030	-0.349; P = 0.421	-2.20; P = 0.20		
RPB2-2	H	678	9	8	0.72	0.66	0.0024	-0.321; P = 0.379	-1.18; P = 0.28	0.15	0.08
bHLH	M	538	22	20	0.83	0.88	0.0041	-1.465; P = 0.043	-9.75; P = 0.00	0.123	
bHLH	C	538	21	19	0.89	0.89	0.0044	-1.280; P = 0.107	-7.85; P = 0.01		
bHLH	H	538	17	15	0.88	0.91	0.0041	-1.335; P = 0.066	-6.44; P = 0.01	0.071	0.003
ANS	M	413	22	25	0.72	0.76	0.0087	-0.510; P = 0.368	-9.72; P = 0.01	0.129	
ANS	C	413	19	20	0.83	0.87	0.0119	0.898; P = 0.858	-2.45; P = 0.23		
ANS	H	413	18	13	0.96	0.90	0.0111	0.378; P = 0.698	-0.68; P = 0.45	0.055	0.009
RPB3	M	512	25	18	0.58	0.60	0.0022	-2.330; P = 0.000	-14.99; P = 0.00	0.189	
RPB3	C	512	16	14	0.81	0.80	0.0040	-0.996; P = 0.175	-3.83; P = 0.09		
RPB3	H	512	14	14	0.74	0.72	0.0026	-1.789; P = 0.018	-9.55; P = 0.00	0.037	0.068
WD40	M	907	20	18	0.72	0.80	0.0036	-0.549; P = 0.337	-4.19; P = 0.08	0.237	
WD40	C	907	12	12	0.85	0.84	0.0018	-0.833; P = 0.217	-3.44; P = 0.08		
WD40	H	907	12	11	0.91	0.88	0.0032	-0.426; P = 0.363	-3.62; P = 0.03	0.087	0.046
WAXY	M	548	20	16	0.59	0.80	0.0043	-1.067; P = 0.163	-6.24; P = 0.01	0.29	
WAXY	C	548	12	10	0.55	0.55	0.0022	-1.333; P = 0.086	-3.26; P = 0.08		
WAXY	H	548	16	14	0.92	0.85	0.0043	-1.067; P = 0.163	-4.76; P = 0.03	0.116	0.067
RPB2-1	M	819	13	13	0.75	0.84	0.0016	-1.287; P = 0.086	-5.63; P = 0.02	0.29	
RPB2-1	C	819	10	10	0.70	0.70	0.0019	-0.5780; P = 0.34	-2.08; P = 0.20		
RPB2-1	H	819	8	9	0.74	0.81	0.0019	-0.478; P = 0.362	-2.60; P = 0.08	0.089	0.097
UF3GT	M	619	18	21	0.76	0.80	0.0034	-1.227; P = 0.096	-12.24; P = 0.01	0.296	
UF3GT	C	619	9	9	0.59	0.74	0.0033	0.393; P = 0.702	-0.18; P = 0.55		
UF3GT	H	619	10	7	0.63	0.70	0.0019	-1.361; P = 0.076	-1.45; P = 0.20	0.287	0.126
F3'H	M	465	33	23	0.66	0.91	0.0097	-0.9260; P = 0.19	-5.00; P = 0.09	0.53	
F3'H	C	465	13	11	0.48	0.50	0.0016	-1.929; P = 0.004	-7.25; P = 0.01		
F3'H	H	465	23	12	0.80	0.81	0.0072	-1.164; P = 0.107	-1.31; P = 0.35	0.185	0.253
MYB	M	598	8	8	0.45	0.60	0.0013	-1.224; P = 0.106	-3.14; P = 0.06	0.714	
MYB	C	598	4	4	0.35	0.37	0.0010	-0.446; P = 0.383	0.05; P = 0.50		
MYB	H	598	3	3	0.84	0.60	0.0025	2.4750; P = 0.99	3.45; P = 0.92	0.323	0.307

[a] *I. cavalcantei* (C), n = 46; *I. marabaensis* (M), n = 48; putative hybrid (H), n = 25.

[b] Analysed gene windows in base pairs (bp).

[c] Number of polymorphic sites in a sample.

[d] Number of haplotypes after SNP phasing.

[e] Observed heterozygosity (He obs); expected heterozygosity (He exp); nucleotide diversity (π).

[f] Cells for pairwise F_{ST} in rows of hybrids (H) is split. The first value is *I. cavalcantei vs* putative hybrids; next value *I. marabaensis vs* putative hybrids. Overall, calculated pairwise F_{ST} of putative hybrids are intermediate as compared species *vs* species, which is in line with expectations for the interspecies hybrids.

Supplementary Table S9. **Analysis of relative rates of synonymous and nonsynonymous substitutions and intragenic recombination.**

			MEME[b,c]	FEL[b,d]			aBRESL[e,f]	BUSTED[e,g]			GARD[h]	
Gene	alleles[a]	sites[b]	pos	pos	neg	n^e	div	ω_1	ω_2	ω_3	(n)	rec
F3'H	33	155	0	1	13	63	0	0.26 (57.6%)	0.61 (42.4%)	1.01 (0%)	820	0
ANS	39	137	3	3	8	75	0	0.38 (76,15%)	0.39 (3.92%)	1.00 (19.92%)	351	0
UF3GT	27	206	0	0	5	51	0	0.36 (87.87%)	0.89 (4.75%)	1.00 (7.38%)	231	0
MYB1	4	111	0	0	0	5	0	0.94 (0%)	1.0 (0%)	1.0 (100%)	112	0
bHLH	15	81	1	1	4	27	0	0.22 (57,39%)	0.46 (42,61%)	1.10 (0%)	120	0
WD40	28	302	0	0	13	53	0	0.01 (87.14%)	0.24 (12.61%)	1.00 (0.25%)	325	1
WAXY	16	122	0	0	4	29	0	0.08 (77.05%)	0.73 (20.74%)	1.00 (2.21%)	120	0
RPB2-1	20	273	0	0	13	37	0	0.00 (3.47%)	0.00 (96.53%)	1.92 (0%)	190	0
RPB2-2	8	124	0	0	4	13	0	0.00 (100%)	0.23 (0%)	2.87 (0%)	45	0
RPB3	6	64	0	0	4	9	0	0.01 (90.06%)	0.20 (9.94%)	1.00(0%)	15	0

[a] Number of haplotypes (alleles) of partial protein-coding gene sequences.

[b] Number of codons (sites) in partial protein-coding gene sequences analysed for the dN/dS (ω) ratio of relative rates of synonymous and nonsynonymous substitutions, using MEME and FEL software.

[c] Mixed Effects Model of Evolution (MEME) software test for the evidence of episodic positive/diversifying selection (pos) with p-value threshold of 0.1.

[d] Fixed Effects Likelihood (FEL) software test for the evidence of pervasive positive/diversifying selection (pos) and pervasive negative/purifying selection (neg) among sites (sites[b]) with p-value threshold of 0.1.

[e] Number of branches of the gene allele phylogenetic trees analysed using aBRESL and BUSTED software.

[f] Adaptive Branch Site REL (aBRESL) software formally tested phylogeny branches (n^e) for diversifying selection (div). Significance was assessed using the Likelihood Ratio Test at a threshold of $p \leq 0.05$, after correcting for multiple testing.

[g] Branch-site Unrestricted Statistical Test for Episodic Diversification (BUSTED) software test for the evidence of gene-wide episodic diversifying selection, i.e. there is an omega > 1, in the selected test branches (n^e) of a given gene phylogeny. Model fits (percentage of model) to a constrained model are shown for the first omega rate class (ω_1), second rate class (ω_2) and third rate class (ω_3).

[h] Genetic Algorithm for Recombination Detection (GARD) software test for recombination (rec) in models (n) with up to 2 breakpoints in analysed genes phylogeny.

Supplementary Table S10. Cluster membership Q-values (admixture coefficient) calculated by STRUCTURE[a].

canga	a/s	sp	colour	Q	type	canga	a/s	sp	colour	Q	type	canga	a/s	sp	colour	Q	type
N1	a	CAV	red	0.9726	WT	N4	s	CAV	purpl	0.9882	WT	N6	a	MAR	lavender	0.0092	WT
N1	a	CAV	red	0.9772	WT	N4	s	CAV	purpl	0.0868	F2/BC	N6	a	MAR	lavender	0.013	WT
N1	a	CAV	red	0.9856	WT	N4	s	H	magenta	0.5566	F1	N6	a	MAR	i-lavender	0.014	WT
N1	a	CAV	red	0.9876	WT	N4	s	H	magenta	0.4114	F1	N6	a	MAR	i-lavender	0.026	WT
N1	a	CAV	red	0.9882	WT	N4	s	H	magenta	0.8654	F2/BC	N6	a	MAR	white	0.011	WT
N1	a	CAV	red	0.99	WT	N4	s	H	magenta	0.4364	F1	N6	a	MAR	white	0.013	WT
N1	a	CAV	red	0.9902	WT	N4	s	H	magenta	0.4086	F1	N7	a	MAR	lavender	0.008	WT
N2	a	CAV	red	0.9812	WT	N4	s	MAR	lavender	0.01	WT	N7	a	MAR	lavender	0.0526	WT
N2	a	CAV	red	0.989	WT	N4	s	MAR	lavender	0.0722	WT	N7	a	MAR	i-lavender	0.0176	WT
N2	a	CAV	red	0.9896	WT	N4	s	MAR	lavender	0.1234	F2/BC	N7	a	MAR	i-lavender	0.0186	WT
N2	a	CAV	red	0.9914	WT	N5	s	CAV	red	0.954	WT	N8	a	MAR	lavender	0.0096	WT
N3	a	CAV	red	0.9786	WT	N5	s	CAV	red	0.9856	WT	N8	a	MAR	lavender	0.01	WT
N3	a	CAV	red	0.9878	WT	N5	s	CAV	red	0.9882	WT	N8	a	MAR	lavender	0.011	WT
N3	a	CAV	red	0.99	WT	N5	s	CAV	pink	0.957	WT	N8	a	MAR	lavender	0.011	WT
N3	a	CAV	red	0.991	WT	N5	s	H	red	0.09	F2/BC	N8	a	MAR	lavender	0.0114	WT
N3	a	CAV	white	0.9912	WT	N5	s	H	magenta	0.2904	F2/BC	N8	a	MAR	lavender	0.0118	WT
N4	s	CAV	red	0.9744	WT	N5	s	H	magenta	0.4828	F1	N8	a	MAR	lavender	0.0122	WT
N4	s	CAV	red	0.9814	WT	N5	s	H	magenta	0.5174	F1	N8	a	MAR	lavender	0.015	WT
N4	s	CAV	red	0.986	WT	N5	s	H	magenta	0.522	F1	N8	a	MAR	lavender	0.0166	WT
N4	s	CAV	red	0.9876	WT	N5	s	H	magenta	0.5538	F1	N8	a	MAR	lavender	0.0268	WT
N4	s	CAV	red	0.9888	WT	N5	s	H	magenta	0.6038	F2/BC	N8	a	MAR	lavender	0.0324	WT
N4	s	CAV	red	0.9898	WT	N5	s	H	magenta	0.6234	F2/BC	N8	a	MAR	lavender	0.371	F2/BC
N4	s	CAV	red	0.99	WT	N5	s	H	magenta	0.6244	F2/BC	S11	a	MAR	lavender	0.0086	WT
N4	s	CAV	red	0.9904	WT	N5	s	H	magenta	0.682	F2/BC	S11	a	MAR	lavender	0.0338	WT
N4	s	CAV	red	0.9904	WT	N5	s	H	magenta	0.6992	F2/BC	S11A	a	MAR	lavender	0.0078	WT
N4	s	CAV	red	0.991	WT	N5	s	H	magenta	0.7012	F2/BC	S11A	a	MAR	lavender	0.008	WT
N4	s	CAV	red	0.991	WT	N5	s	H	magenta	0.716	F2/BC	S11A	a	MAR	lavender	0.008	WT
N4	s	CAV	red	0.992	WT	N5	s	H	magenta	0.726	F2/BC	S11A	a	MAR	lavender	0.0094	WT
N4	s	CAV	red	0.993	WT	N5	s	H	magenta	0.82	F2/BC	S11A	a	MAR	lavender	0.0124	WT
N4	s	CAV	pink	0.9796	WT	N5	s	H	red	0.9058	WT	S11A	a	MAR	lavender	0.0136	WT
N4	s	CAV	pink	0.9774	WT	N5	s	H	red	0.9226	WT	S11A	a	MAR	lavender	0.0146	WT
N4	s	CAV	pink	0.986	WT	N5	s	H	purple	0.973	WT	S11D	a	MAR	lavender	0.007	WT
N4	s	CAV	pink	0.9888	WT	N5	s	H	magenta	0.5808	F1	S11D	a	MAR	lavender	0.0096	WT
N4	s	CAV	pink	0.9644	WT	N5	s	H	magenta	0.7054	F2/BC	S11D	a	MAR	lavender	0.01	WT
N4	s	CAV	pink	0.9388	WT	N5	s	MAR	lavender	0.017	WT	S11D	a	MAR	lavender	0.0142	WT
N4	s	CAV	pink	0.9914	WT	N5	s	MAR	lavender	0.2018	F2/BC	SO	a	MAR	lavender	0.0084	WT
N4	s	CAV	purpl	0.9722	WT	N5	s	MAR	pink	0.0414	WT	SO	a	MAR	lavender	0.0132	WT
N4	s	CAV	purpl	0.884	F2/BC	N5	s	MAR	pink	0.1134	F2/BC	TARZAN	a	MAR	lavender	0.009	WT
N4	s	CAV	purpl	0.992	WT	N5	s	MAR	pink	0.5756	F1	TARZAN	a	MAR	lavender	0.0128	WT
N4	s	CAV	purpl	0.9128	WT	N6	a	MAR	lavender	0.008	WT						

[a] Columns are: (canga) - the origin of individuals from canga; (a/s) – allopatric cangas (a), or cangas with sympatry (s); (sp) – *I. cavalcantei* (CAV), *I. marabaensis* (MAR), putative hybrids (H); (colour) – indicates flower colour, here (purpl is purplish; i-lavender, intense lavender); (Q) – admixture coefficient; (type) – type of individual classified by the Q thresholds as follows: Q < 0.1 wild type *I. marabaensis*, or > 0.9 wild type *I. cavalcantei* (WT); Q in a range 0.4-0.6, F1 hybrid (F1); Q in ranges 0.1-0.4 or 0.6-0.9, F2/backcross progeny (F2/BC).

Supplementary Table S11. Pollen viability staining.

Canga origin	Collection[a]	Taxon[b]	Flower colour [c]	ID[d]	Replicates[e]	Pollen grains[f]	Viable	%[g]	Aborted	%
N4	*ex situ*	H	magenta	EB088	#1	300	282	94.0	18	6.0
N4	*ex situ*	H	magenta	EB088	#2	300	277	92.3	23	7.7
N4	*ex situ*	H	magenta	EB088	#3	300	299	99.7	1	0.3
								AV=95.3		
N4	*ex situ*	H	magenta	EB081	#1	300	299	99.7	1	0.3
N4	*ex situ*	H	magenta	EB081	#2	310	297	95.8	13	4.2
N4	*ex situ*	H	magenta	EB081	#3	300	298	99.3	2	0.7
								AV=98.3		
N4	*ex situ*	H	magenta	EB022	#1	300	287	95.7	13	4.3
N4	*ex situ*	C	purplish	EB123	#1	300	295	98.3	5	1.7
N4	*ex situ*	C	purplish	EB123	#2	300	288	96.0	12	4.0
N4	*ex situ*	C	purplish	EB123	#3	300	298	99.3	2	0.7
								AV=97.9		
N4	*ex situ*	C	pink	EB139	#1	300	297	99.0	3	1.0
N4	*ex situ*	C	pink	EB139	#2	300	296	98.7	4	1.3
N4	*ex situ*	C	pink	EB139	#3	300	296	98.7	4	1.3
								AV=98.8		
N4	*ex situ*	C	pink	EB079	#1	300	297	99.0	3	1.0
N4	*ex situ*	C	pink	EB017	#1	300	292	97.3	8	2.7
N1	*in situ*	C	red	PL1	#1	300	262	87.3	38	12.7
N1	*in situ*	C	red	PL2	#1	300	297	99.0	3	1.0
N1	*in situ*	C	red	PL3	#1	300	293	97.7	7	2.3
N1	*in situ*	C	red	PL4	#1	300	297	99.0	3	1.0
N3	*in situ*	C	red	PL1	#1	300	291	97.0	9	3.0
N3	*in situ*	C	red	PL2	#1	300	297	99.0	3	1.0
N3	*in situ*	C	red	PL3	#1	300	291	97.0	9	3.0
N3	*in situ*	C	red	PL4	#1	300	287	95.7	13	4.3
								AV=96.5		
N4	*ex situ*	M	pale lavender	EB023	#1	300	300	100.0	0	0.0
N4	*ex situ*	M	pale lavender	EB023	#2	300	297	99.0	3	1.0
								AV=99.5		
N6	*ex situ*	M	white	EB024	#1	300	297	99.0	3	1.0
N6	*ex situ*	M	white	EB024	#2	300	296	98.7	4	1.3
								AV=98.8		
N7	*in situ*	M	pale lavender	PL1	#1	300	290	96.7	10	3.3
N7	*in situ*	M	pale lavender	PL2	#1	300	288	96.0	12	4.0
N7	*in situ*	M	pale lavender	PL3	#1	300	298	99.3	2	0.7
N8	*in situ*	M	pale lavender	PL1	#1	300	297	99.0	3	1.0
N8	*in situ*	M	pale lavender	PL2	#1	300	285	95.0	15	5.0
								AV=97.2		

[a] Pollen viability was measured using flower buds one day before anthesis collected either from plants in *ex situ* collection (*ex situ*) or from plants in a field (*in situ*).
[b] The taxon are *I. cavalcantei* (C); *I. marabaensis* (M) or putative hybrids (H).
[c] The colour of flower limb that characterize flowers from an individual is indicated.

[d] Plants in *ex situ* collection all had an identifier number, e.g. EB088. Plants from *in situ* sampling were randomly chosen, e.g. PL1, PL2 etc. individual, a single flower per plant was analysed.
[e] Depending on flower availability from individuals in *ex situ* collection, several independent flower buds were analysed as experimental replicates.
[f] The number of pollen grains counted per sample, mostly 300, except one sample of 310 grains.
[g] For the replicated samples per individuals from *ex situ* collection the average for a plant is shown (AV). The average (AV) with *in situ* sampling is for all plants from a given canga.

Supplementary Table S12. Seed production by putative hybrids after controlled hand-pollinations[a].

Style[b]	Pollen[b]	Flower[c]	Cross[d]	Seeds[e]	Parental combinations total/compatible[f]	Seed set (%)[g]
EB081 (H/M/N4)	CAVN4 (C/R/N4)	CAPSULE	H×C	4	5/4	75
EB081 (H/M/N4)	CAVPZB2 (C/R/N4)	ABORTED	H×C	0		
EB081 (H/M/N4)	CAVPZB3 (C/R/N4)	CAPSULE	H×C	3		
EB081 (H/M/N4)	EB139 (C/P/N4)	CAPSULE	H×C	2		
EB081 (H/M/N4)	EBD1 (C/R/N4)	CAPSULE	H×C	3		
EB081 (H/M/N4)	EB156 (M/L/N8)	CAPSULE	H×M	3	9/6	82
EB081 (H/M/N4)	EBPZB1 (M/L/S11)	ABORTED	H×M	0		
EB081 (H/M/N4)	EBPZB1 (M/L/S11)	ABORTED	H×M	0		
EB081 (H/M/N4)	MARN6 (M/L/N6)	CAPSULE	H×M	4		
EB081 (H/M/N4)	MAR_N5-2 (M/L/N5)	CAPSULE	H×M	4		
EB081 (H/M/N4)	MAR_N5-3 (M/L/N5)	ABORTED	H×M	0		
EB081 (H/M/N4)	MAR_N7-1 (M/L/N7)	ABORTED	H×M	0		
EB081 (H/M/N4)	MAR_N7-2 (M/L/N7)	CAPSULE	H×M	2		
EB081 (H/M/N4)	MAR_N8-1 (M/L/N8)	CAPSULE	H×M	2		
EB081 (H/M/N4)	MAR_N8-1 (M/L/N8)	CAPSULE	H×M	4		
EB081 (H/M/N4)	MAR_N8-2 (M/L/N8)	CAPSULE	H×M	4		
EB081 (H/M/N4)	EB015 (H/M/N4)	CAPSULE	H×H	3	4/2	66
EB081 (H/M/N4)	EB015 (H/M/N4)	CAPSULE	H×H	3		
EB081 (H/M/N4)	EB015 (H/M/N4)	CAPSULE	H×H	4		
EB081 (H/M/N4)	EB015 (H/M/N4)	CAPSULE	H×H	3		
EB081 (H/M/N4)	EB015 (H/M/N4)	CAPSULE	H×H	2		
EB081 (H/M/N4)	EB015 (H/M/N4)	CAPSULE	H×H	1		
EB081 (H/M/N4)	EB015 (H/M/N4)	CAPSULE	H×H	2		
EB081 (H/M/N4)	EB015 (H/M/N4)	CAPSULE	H×H	3		
EB081 (H/M/N4)	EB015 (H/M/N4)	CAPSULE	H×H	2		
EB081 (H/M/N4)	EB015 (H/M/N4)	CAPSULE	H×H	2		
EB081 (H/M/N4)	EB015 (H/M/N4)	CAPSULE	H×H	4		
EB081 (H/M/N4)	EB015 (H/M/N4)	ABORTED	H×H	0		
EB081 (H/M/N4)	EB015 (H/M/N4)	ABORTED	H×H	0		
EB081 (H/M/N4)	EB088 (H/M/N4)	ABORTED	H×H	0		
EB081 (H/M/N4)	EB088 (H/M/N4)	ABORTED	H×H	0		
EB081 (H/M/N4)	EB161 (H/M/N5)	CAPSULE	H×H	3		
EB081 (H/M/N4)	EB161 (H/M/N5)	CAPSULE	H×H	4		
EB081 (H/M/N4)	EB161 (H/M/N5)	CAPSULE	H×H	2		
EB081 (H/M/N4)	EB161 (H/M/N5)	CAPSULE	H×H	2		
EB081 (H/M/N4)	EB161 (H/M/N5)	CAPSULE	H×H	3		
EB081 (H/M/N4)	EB161 (H/M/N5)	CAPSULE	H×H	2		
EB081 (H/M/N4)	EB161 (H/M/N5)	CAPSULE	H×H	4		
EB081 (H/M/N4)	EB161 (H/M/N5)	CAPSULE	H×H	1		
EB081 (H/M/N4)	EB161 (H/M/N5)	CAPSULE	H×H	3		
EB081 (H/M/N4)	EB162 (H/M/N5)	CAPSULE	H×H	0		

EB081 (H/M/N4)	EB162 (H/M/N5)	CAPSULE	H×H	0		
EB081 (H/M/N4)	EB162 (H/M/N5)	CAPSULE	H×H	0		
EB081 (H/M/N4)	EB162 (H/M/N5)	CAPSULE	H×H	0		
EB123 (C/U/N4)	EB081 (H/M/N4)	ABORTED	C×H	0	3/1	100
EB139 (C/P/N4)	EB081 (H/M/N4)	CAPSULE	C×H	4		
EB148 (C/R/N4)	EB081 (H/M/N4)	ABORTED	C×H	0		
EB129 (M/L/N6)	EB081 (H/M/N4)	CAPSULE	M×H	3	1/1	75
EB022 (H/M/N4)	CAV_PZB2 (C/R/N4)	CAPSULE	H×C	4	4/2	100
EB022 (H/M/N4)	CAV_PZB3 (C/R/N4)	ABORTED	H×C	0		
EB022 (H/M/N4)	CAV_PZB4 (C/R/N4)	ABORTED	H×C	0		
EB022 (H/M/N4)	CAV_PZB5 (C/R/N4)	CAPSULE	H×C	4		
EB022 (H/M/N4)	MAR_N5-1 (M/L/N5)	CAPSULE	H×M	4	2/2	100
EB022 (H/M/N4)	MAR_N8-2 (M/L/N8)	CAPSULE	H×M	4		
EB022 (H/M/N4)	EB015 (H/M/N4)	CAPSULE	H×H	4	1/1	100
EB048 (C/P/N4)	EB022 (H/M/N4)	CAPSULE	C×H	4	6/3	100
EB123 (C/U/N4)	EB022 (H/M/N4)	ABORTED	C×H	0		
EB139 (C/P/N4)	EB022 (H/M/N4)	CAPSULE	C×H	4		
EB079 (C/P/N4)	EB022 (H/M/N4)	CAPSULE	C×H	4		
EB079 (C/P/N4)	EB022 (H/M/N4)	CAPSULE	C×H	4		
EB079 (C/P/N4)	EB022 (H/M/N4)	CAPSULE	C×H	4		
EB079 (C/P/N4)	EB022 (H/M/N4)	CAPSULE	C×H	4		
EBD1 (C/R/N4)	EB022 (H/M/N4)	ABORTED	C×H	0		
EBD1 (C/R/N4)	EB022 (H/M/N4)	ABORTED	C×H	0		
EBD1 (C/R/N4)	EB022 (H/M/N4)	ABORTED	C×H	0		
EBD2 (C/R/N4)	EB022 (H/M/N4)	ABORTED	C×H	0		
EB129 (M/L/N6)	EB022 (H/M/N4)	ABORTED	M×H	0	1/0	NA
EB129 (M/L/N6)	EB022 (H/M/N4)	ABORTED	M×H	0		
EB048 (C/P/N4)	EB015 (H/M/N4)	CAPSULE	C×H	4	4/4	70
EB048 (C/P/N4)	EB015 (H/M/N4)	CAPSULE	C×H	3		
EB139 (C/P/N4)	EB015 (H/M/N4)	CAPSULE	C×H	1		
EB148 (C/R/N4)	EB015 (H/M/N4)	CAPSULE	C×H	4		
EB079 (C/P/N4)	EB015 (H/M/N4)	CAPSULE	C×H	2		
EB048 (C/P/N4)	EB088 (H/M/N4)	CAPSULE	C×H	4	4/3	88
EB048 (C/P/N4)	EB088 (H/M/N4)	CAPSULE	C×H	3		
EB139 (C/P/N4)	EB088 (H/M/N4)	CAPSULE	C×H	4		
EB148 (C/R/N4)	EB088 (H/M/N4)	CAPSULE	C×H	3		
EB079 (C/P/N4)	EB088 (H/M/N4)	ABORTED	C×H	0		
EB129 (M/L/N6)	EB088 (H/M/N4)	ABORTED	M×H	0	1/0	NA
EB129 (M/L/N6)	EB088 (H/M/N4)	ABORTED	M×H	0		
EB129 (M/L/N6)	EB088 (H/M/N4)	ABORTED	M×H	0		

[a] All putative hybrids individuals are perennials that enter dormancy during dry season (June-December). Four tested putative hybrids EB015; EB022; EB081; EB088 were rescued from canga N4 and grown in *ex situ* collection at VALE Zoo-botanical park (Carajás, Pará). Tested putative hybrids were self-incompatible (Supplementary Table S5). Flowers availability was

one limiting factor for the number of crosses we can perform. Only EB081 flowered abundantly during wet season January-May 2018. Plants with identification numbers beginning with "EB" are from ex situ collection. Other identifications, such as "CAV" or "MAR" are for the flowers collected from respective canga in the wild from different individuals.

[b] The basic information about individual plants is summarized in parentheses beginning with C, or M or H, which are hybrid (H); *I. cavalcantei* (C); *I. marabaensis* (M), respectively. The next letter describes the flower colour: magenta (M); lavender (L); red (R); pink (P); purplish (U). Next are the canga of origin: N4, N5, N6, N7, N8 and S11 Plateau (S11).
[c] The fate of pollinated flower, either aborted within a week (ABORTED) or developed into a fruit capsule (CAPSULE).
[d] Type of cross and direction "mother × father".
[e] Number of seeds per capsule, expected four.
[f] Summary of crosses. The number of tested parental combinations is the first number, followed, after a slash, by the number of parental combinations that resulted in seed development.
[g] Percentage of productive fertilizations events from the expected events of four per fruit capsule.

Supplementary Table S13. Interspecies hybridization by hand-pollinations.

Summary of interspecific crosses[a]				
**Cross[a]**	**Parental combinations total/compatible**	**Flower/fruits**	**Seeds Exp/actual**	**Seed set**
C×M	27/11	27/11	44/35	80%
M×C	9/6	10/7	28/24	85%
**List of individual interspecific crosses[b]**				
**Style**	**Pollen**	**Flower**	**Cross**	**Seeds**
EB048 (C/P/N4)	MAR_N5-5 (M/L/N5)	ABORTED	C×M	0
EB123 (C/U/N4)	MAR_N7-1 (M/L/N7)	CAPSULE	C×M	4
EB139 (C/P/N4)	EBPZB1 (M/L/S11)	ABORTED	C×M	0
EB139 (C/P/N4)	MAR_N5-1 (M/L/N5)	CAPSULE	C×M	4
EB139 (C/P/N4)	MAR_N5-2 (M/L/N5)	ABORTED	C×M	0
EB139 (C/P/N4)	MAR_N5-3 (M/L/N5)	ABORTED	C×M	0
EB139 (C/P/N4)	MAR_N5-4 (M/L/N5)	ABORTED	C×M	0
EB139 (C/P/N4)	MAR_N7-1 (M/L/N7)	ABORTED	C×M	0
EB139 (C/P/N4)	MAR_N7-2 (M/L/N7)	ABORTED	C×M	0
EB147 (C/U/N4)	EBPZB1 (M/L/S11)	ABORTED	C×M	0
EB148 (C/R/N4)	EB156 (M/L/N8)	CAPSULE	C×M	4
EB148 (C/R/N4)	MAR_N5-2 (M/L/N5)	ABORTED	C×M	0
EB148 (C/R/N4)	MAR_N7-2 (M/L/N7)	ABORTED	C×M	0
EB079 (C/P/N4)	MAR_N5-1 (M/L/N5)	CAPSULE	C×M	2
EBD1 (C/R/N4)	EB024 (M/L/N6)	CAPSULE	C×M	3
EBD1 (C/R/N4)	EB156 (M/L/N8)	CAPSULE	C×M	4
EBD1 (C/R/N4)	EBPZB1 (M/L/S11)	ABORTED	C×M	0
EBD1 (C/R/N4)	EBPZB2 (M/L/N8)	CAPSULE	C×M	3
EBD1 (C/R/N4)	MAR_N5-1 (M/L/N5)	ABORTED	C×M	0
EBD1 (C/R/N4)	MAR_N5-2 (M/L/N5)	CAPSULE	C×M	4
EBD1 (C/R/N4)	MAR_N5-3 (M/L/N5)	CAPSULE	C×M	1
EBD1 (C/R/N4)	MAR_N5-4 (M/L/N5)	ABORTED	C×M	0
EBD1 (C/R/N4)	MAR_N5-5 (M/L/N5)	CAPSULE	C×M	2
EBD1 (C/R/N4)	MAR_SO1 (M/L/SO)	CAPSULE	C×M	4
EBD1 (C/R/N4)	MAR_SO2 (M/L/SO)	ABORTED	C×M	0
EBD2 (C/R/N4)	EB156 (M/L/N8)	ABORTED	C×M	0
EBD2 (C/R/N4)	MAR_N5-5 (M/L/N5)	ABORTED	C×M	0
EB024 (M/W/N6)	CAV_N4 (C/R/N4)	CAPSULE	M×C	4
EB024 (M/W/N6)	CAV_PZB1 (C/R/N4)	CAPSULE	M×C	0
EB024 (M/W/N6)	CAV_PZB3 (C/R/N4)	CAPSULE	M×C	4
EB024 (M/W/N6)	EBD1 (C/R/N4)	CAPSULE	M×C	4
EB024 (M/W/N6)	EBD1 (C/R/N4)	CAPSULE	M×C	4
EB129 (M/L/N6)	CAV_N3-1 (C/R/N3)	CAPSULE	M×C	3
EB129 (M/L/N6)	CAV_PZB2 (C/R/N4)	CAPSULE	M×C	3
EB129 (M/L/N6)	CAV_PZB3 (C/R/N4)	ABORTED	M×C	0
EB129 (M/L/N6)	CAV_PZB4 (C/R/N4)	CAPSULE	M×C	0

EB129 (M/L/N6)	EBD2 (C/R/N4)	CAPSULE	M×C	2

[a] Part of the Table that summarizes the results individual crosses. Crosses were *I. cavalcantei* × *I. marabaensis* (C×M); *I. marabaensis* × *I. cavalcantei* (M×C).

[b] All ovule donors were from *ex situ* collection at VALE Zoobotanical park, including: *I. marabaensis* EB024 and EB129 individuals from canga N6; *I. cavalcantei* from canga N4, i.e. three red-flowered individuals, three pink flower plants EB079, EB048, EB0139, one purplish EB123. Pollen was collected either from plants in *ex situ* collection, or from *I. marabaensis* plants in canga N5, N8, inselberg Sossego (SO). *I. marabaensis* pollen from a wild was used both because of limited number of individuals in *ex situ* collection, and to test the effect of canga separation by distance on fertilization success, which could have been underpinned by stronger genetic differentiation due to geographic isolation, e.g. canga N8 and Sossego (SO). Other abbreviations and column identifiers as in the Supplementary Table S12.

Supplementary Table S14. Nectar production by the isolated flowers in *ex situ* collection.

Individuals[a]	Species; flower colour[b]	flower	hour of collection	Nectar volume, µL
EBD1	C; red	#1	17:00 PM	50
EBD1	C; red	#2	16:30 PM	90
EBD1	C; red	#3	17:00 PM	55
EBD1	C; red	#4	16:30 PM	46
EBD1	C; red	#5	16:30 PM	35
EBD1	C; red	#6	16:30 PM	90
EBD1	C; red	#7	16:30 PM	80
EBD1	C; red	#8	16:30 PM	65
EBD1	C; red	#9	16:30 PM	60
EB146	C; red	#1	17:00 PM	60
EB146	C; red	#2	17:00 PM	80
EB048	C; pink	#1	16:30 PM	80
EB048	C; pink	#2	16:30 PM	80
EB048	C; pink	#3	16:30 PM	30
				AV 64
EBPZB1	M; lavender	#1	16:00 PM	78
EBPZB1	M; lavender	#2	16:00 PM	70
EBPZB2	M; pale lavender	#1	15:30 PM	60
EBPZB3	M; pale lavender	#1	15:30 PM	90
				AV 75
EB015	H; magenta	#1	15:30 PM	55
EB022	H; magenta	#1	17:00 PM	79
EB022	H; magenta	#2	16:30 PM	70
EB022	H; magenta	#3	17:00 PM	70
EB081	H; magenta	#1	17:00 PM	90
EB081	H; magenta	#2	16:30 PM	80
EB081	H; magenta	#3	17:00 PM	111
EB081	H; magenta	#4	16:30 PM	60
EB081	H; magenta	#5	16:30 PM	80
EB081	H; magenta	#6	16:30 PM	70
EB088	H; magenta	#1	17:00 PM	88
EB088	H; magenta	#2	16:30 PM	80
				AV 77

[a] All measurements are from the plants in *ex situ* collection. To prevent nectar losses due to nectar robbing or consumption by pollinators, flower buds were isolated at one day before anthesis. Next day, bagged isolated flowers were cut off the plant at indicated hours and nectar volume (in µL) was measured by withdrawing all the liquid accumulated within and above nectar chamber. Nectar samples that were of smaller volume than the species average was also denser, as visualized by mixing samples in between, indicating that sugar concentration varied both within and in between individuals. We did not analyse the nectar production by the flower buds. Since *I. cavalcantei* flowers open as early as 5:00 am, the shown nectar volumes were

produced by approximately 12 hours-old flowers at anthesis. Average values (AV). In *Ipomoea* flowers, the bases of five filaments bulge out from the adaxial corolla surface touching style. Those morphological features separate the proximal tube space, forming what is known as a nectar chamber. Five triangular openings to nectar chamber are small, ca. 1 mm at widest side in *I. cavalcantei* and *I. marabaensis*, and are further occluded by well-developed trichomes, likely discriminating nectar foragers by the dimensions of their tongues and other mouth parts. In some flowers, nectar can overspill from the chamber into the tube proper. This nectar fraction is more easily accessible to visitors such as beetles or wasps, which were numerous. The flower calix, a whorl of sepals, covers the corolla tube parts that comprise nectar chamber.
[b] *I. cavalcantei* (C); *I. marabaensis* (M), colour variants (pink) and interspecies hybrids (H; magenta) were analysed.

Supplementary Table S15. Nectar production by non-isolated *I. cavalcantei* flowers collected in Canga N1.

Plant	Collection time	Measurement time	Beetles in the tubes[b]	Nectar volume, μL
#1	7:30 AM	14:10 PM	YES	30
#2	7:30 AM	14:10 PM	YES	40
#3	7:30 AM	14:10 PM	NO	35
#4	7:30 AM	14:10 PM	NO	20
#5	7:30 AM	14:10 PM	NO	90
#6	7:30 AM	14:10 PM	YES	20
#7	7:30 AM	14:10 PM	NO	47
#8	7:30 AM	14:10 PM	NO	75
#9	7:30 AM	14:10 PM	YES	30
#10	7:30 AM	14:10 PM	YES	42
#11	7:30 AM	14:10 PM	YES	0
#12	7:30 AM	14:10 PM	NO	20
			50% of flowers	AV 37

[a] Flowers from twelve different *I. cavalcantei* plants were collected in Canga N1. Flowers were placed in plastic tubes filled with water to just cover the flower peduncle for water supply and maintenance of turgor. After the transport to the laboratory, nectar volumes were measured in the afternoon, ca. 7 hours after collection in a wild. Average value (AV) is shown in the bottommost row. The difference in average here as compared to Supplementary Table 11, suggests that either some nectar has been taken by the flower visitors between 5:00 am and 7:30 am, or/and physiological differences between plants from canga and plants in *ex situ* collection that is positioned on land cleared from rain forest. It was not feasible to bag flower buds in wild populations.

[b] This column indicates whether beetles were present (YES or NO) in the flower at the time of nectar measurement. The species of beetles (not identified in this work, body length ca. 1 cm) were commonly found in the tubes of *I. cavalcantei* flowers. It is unlikely that the architecture of beetle mouth is suitable for the animal to reach nectar concealed within nectar chamber. Beetles often aggregated up to ten individuals in a single flower tube, possibly mating. Flower tube is also a good place to avoid predation. We did not find indications that *I. cavalcantei* beetles were feeding of flower tissues. This is different from *I. marabaensis* flowers in which smaller (2-5 mm body length) species (at least 4 species) of beetles were very common. Beetle infestation correlated with flower tube discoloration due to animal grazing on adaxial flower tissues, which however did not lead to perforations in a tube.

Supplementary Table S16. Estimates of honeybee visitation frequencies on *I. cavalcantei*.

Individuals	Flowers within videoframe	Total movie footage, seconds	Honeybee visits	Frequency of visits per plant, seconds
Plant#1	6	567	11	51
Plant#2	9	405	8	50

[a] Short (23 to 370 seconds) movies of two *I. cavalcantei* individuals were taken within 50 min (Plant#1) and 40 min (Plant#2) in the morning of the same day. Plants were about 50 meters apart. The total video footage is a combined duration of twelve (Plant#1) and two (Plant#2) movies. Honeybee entering the tubes of observed within the videoframe flowers were counted as “honeybee visits”. The frequency of visits per plant was inferred by dividing the total footage duration by the number of visits. The result suggests that at this location (canga N1), *I. cavalcantei* individuals were visited by at least one honeybee every minute. Since we did not know the locations of honeybee nests, we did not elaborate further on that type of measurements in presented field work.

Supplementary Table S17. Interactions of honeybees with *I. cavalcantei* flowers.

Flowers[a]	Exit time groups[b]			Stigma-bee contact[c]		
	easy 1-2 s	difficult 3-30 s	traps > 30 s	yes	no	NA
136	26	73	37	34	73	29
	19%	54%	27%	32%	68%	NA

[a] 136 video clips were examined to characterize interactions between honeybees and different flowers at relatively easy to access and least disturbed canga N1.
[b] Bee exit time was measured in seconds (s) as a time elapsed from the appearance of bee abdomen in flower tube opening (throat) to the appearance of animal head. The exit times varied from 1 second to several hours, therefore we grouped exit times as “easy”; “difficult” and “traps”.
[c] The contact of bee body with stigma is a critical requirement for the pollen transfer. Not applicable (NA) refers to cases when either bee contact with stigma was not well visualized in a given clip, or when flowers had no stigmas (nine flowers). The nectar robbing by *Trigona* spp. bees, that routinely removed reproductive organs from *I. cavalcantei* flowers, (Supplementary Figure S7) is the most likely reason for the absence of styles and stigmas.

Supplementary Table S18. Oligonucleotide primer sequences.

Primer ID	Oligonucleotide sequence 5' to 3'	Amplicon ID[a]	Gene
HLH_DWN_F2	GAATGGGGAGAAACTGTAAGGACTGTGA	PC18	*bHLH*
BHLHF	ATGGCAAGGGGAGAGGGATTTGCAGA	PC18	*bHLH*
HYDSF1	CAAGACCCATCCCACGAAAGGTTAAA	PC10	*F3'H*
HYDE2R3	CATGGGATGTTAGGGAATACCAACCAA	PC10	*F3'H*
PCR23BF2	ACAGGACACATTGGCCTATGTGCTA	PC23	*RPB2-1*
PCR23BSEQR2	ATCCACCATGTGCTTCAGCCTT	PC23	*RPB2-1*
PCR23AF1	CCGCTTTTCTGACTGATATATTGTTGTGCA	PC24	*RPB2-2*
PCR23ASEQR2	GAATCGTGGATCAGGTTCACAATAC	PC24	*RPB2-2*
RPB3-FWD1	AGCGAGCGCGCYATGAGCATGCG	PC16	*RPB3*
RPB3NR1	CCTTAGCTAGTTTGAAGCCAAATGACA	PC16	*RPB3*
WAX_F1	ATGGATACCCAAGAGTGGAACCCTGCAACT	PC15	*WAXY*
WAX_R1	TCGCATAGCATGCAACTGAATGAGACCACA	PC15	*WAXY*
ANSP32F-RAUSHER	CAACTGTTCCCAGCAGGGTG	PCR20	*ANS*
ANSR1	GGGACCATGTTGTGGAGGATGAA	PCR20	*ANS*
MYBF	TGCATTCAGAAATTTGGTGAAGGAAAATGG	PCR2	*MYB*
MYBR1	CTCCTAGGTCGAGGCYTAA	PCR2	*MYB*
UFGT-F1	CATCATGGCGATGCCGGGTAATTA	PCR4	*UF3GT*
UFGT-R2	AAGGGACCAGAGAAAGGGTGC	PCR4	*UF3GT*
WD40F	ATGGAGAACTCAACCCAAGGATCGAA	PCR10	*WD40*
WD40R	TTACACTTTTAGCATCTGAAGCTT	PCR10	*WD40*

[a] Amplicons were produced on a thermocycler programmed to execute the following steps: (1) 94°C 2 min; (2) five T_m-step-down cycles: 94°C 15 seconds; 65°C minus 2°C per cycle, 30 sec; 72°C 60 sec; (3) 30 amplification cycles: 94°C 15 sec; 58°C 30 sec; 72°C sec plus 2 sec per cycle, 60 sec; (4) extension step, 72°C 10 min; (5) storage step 4°C, indefinite time.
